# Habitat fragmentation can either increase or decrease with habitat loss

**DOI:** 10.1007/s10980-026-02345-8

**Published:** 2026-04-09

**Authors:** Amanda E. Martin, Carmen Galán-Acedo, Federico Riva, Lenore Fahrig

**Affiliations:** 1 Environment and Climate Change Canada, National Wildlife Research Centre, Ottawa, ON Canada; 2 Department of Biology, Carleton University, Ottawa, ON Canada; 3 Department of Environmental Geography, Institute for Environmental Studies (IVM), VU Amsterdam, Amsterdam, The Netherlands

**Keywords:** Forest fragmentation index, Fragmented landscape, Fragmentation per se, Landscape configuration, Landscape fragmentation, Landscape pattern

## Abstract

**Context:**

A key aspect of the fragmentation debate in conservation biology is whether fragmentation effects on biodiversity should capture the combined or separate effects of habitat loss and fragmentation, i.e., breaking apart of habitat into multiple patches. A common argument for treating loss and fragmentation as a single phenomenon is that human-caused habitat loss almost always leads to fragmentation.

**Objectives:**

Here we assessed whether forest loss consistently results in fragmentation, at a global extent and across spatial scales commonly considered in landscape ecology.

**Methods:**

We evaluated how often forest loss resulted in a decrease versus increase in fragmentation for 150,000 randomly-selected forest locations. We delineated landscapes of six sizes (radii of 0.25 to 10 km) at each location. For the subset of landscapes that lost forest between 2000 and 2020, we estimated the change in fragmentation using four different measures of fragmentation.

**Results:**

A decrease in forest fragmentation was a common outcome of forest loss. Across four measures of fragmentation, six landscape sizes, and all forested biomes, we found forests were more fragmented after forest loss 51% of the time and less fragmented 44% of the time.

**Conclusions:**

Our results show that the fact that fragmentation often results from habitat loss does not mean that fragmentation is the inevitable consequence of habitat loss. Effects of habitat loss on biodiversity often differ from those of fragmentation separate from habitat amount (fragmentation per se). Thus, understanding the effects of fragmentation per se on biodiversity is not only feasible, it is fundamental for developing effective habitat conservation plans to address biodiversity loss.

**Supplementary Information:**

The online version contains supplementary material available at 10.1007/s10980-026-02345-8.

## Introduction

Habitat fragmentation has been one of the most important—and debated—topics in conservation biology (Miller-Rushing et al. 2019). This debate centers on whether empirical evidence supports the widespread expectation that fragmentation should be “bad” for biodiversity (Fahrig 2017; Fletcher et al. 2018; Fahrig et al. 2019; Valente et al. 2023). Some studies find that ecological responses (e.g., species richness, abundance) are lower in landscapes with less habitat fragmented into more patches than in landscapes with more habitat and fewer patches (e.g., Haddad et al. 2015). However, studies that control for the total area of habitat typically find that ecological responses are the same or even higher in landscapes with a given amount of habitat fragmented into more (smaller) patches than in landscapes with fewer (larger) patches (reviewed in Fahrig 2017). Thus, a key distinction between studies concluding that habitat fragmentation reduces biodiversity, and those concluding the opposite, can be whether the authors conceptualize habitat fragmentation as inherently combined with habitat loss.

**Fig. 1:**
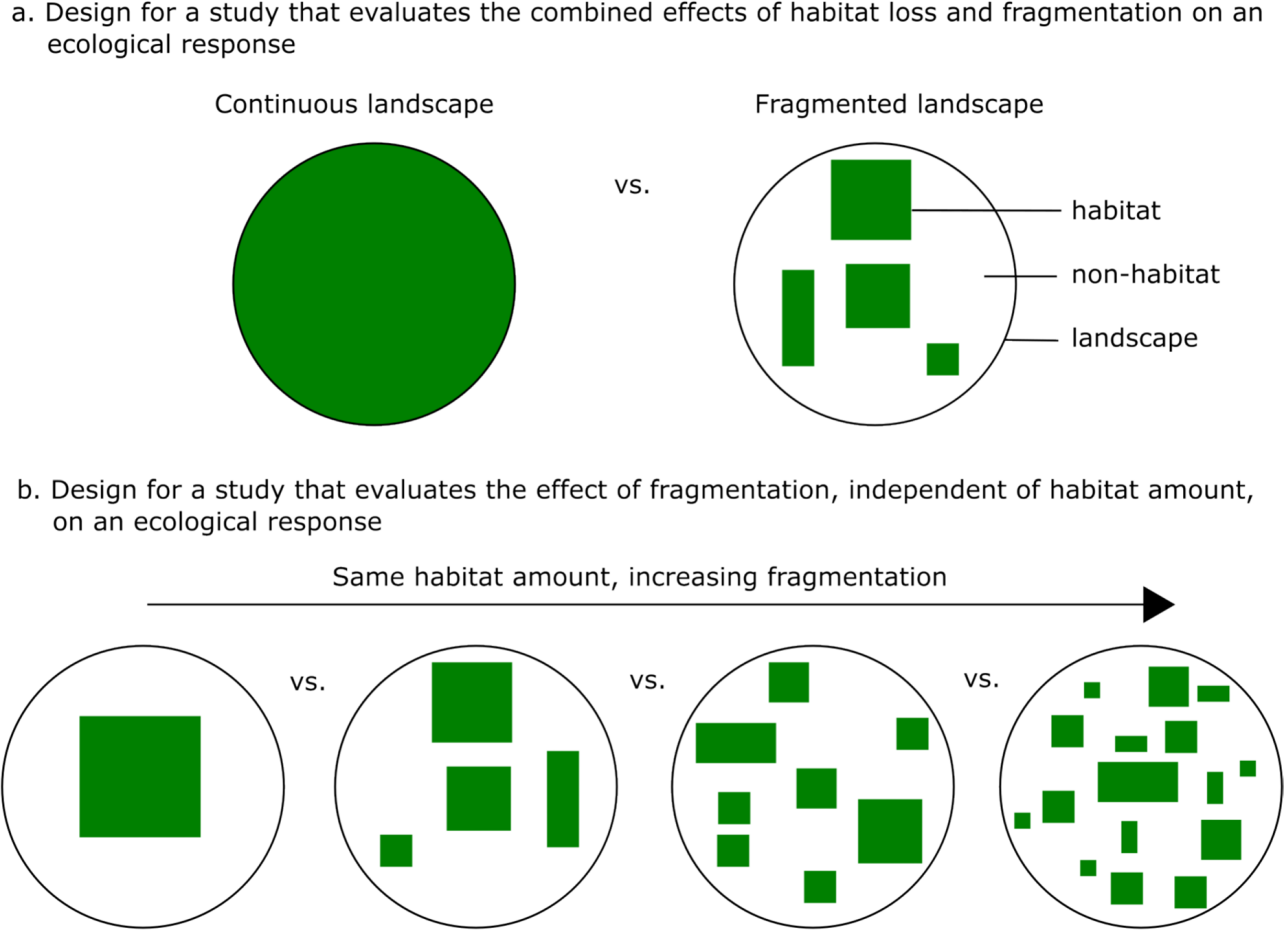
**a** An illustrative example of the typical design in studies that evaluate the combined effects of habitat loss and fragmentation on an ecological response. A “continuous” landscape (with complete, or near-complete, habitat cover) is paired with a “fragmented” landscape (with less habitat, subdivided into multiple patches). The paired landscapes are meant to represent a given landscape before and after removal of habitat. **b** An illustrative example of the typical design in studies that evaluate the effects of habitat fragmentation on an ecological response, independent of habitat amount. Multiple landscapes are compared, with each landscape meant to represent an alternative endpoint of a given level of habitat loss. This is done by selecting landscapes with the same habitat amount but different levels of fragmentation (as shown here), or by statistically controlling for variation in habitat amount across landscapes

Researchers who conclude that fragmentation effects are negative often use the classical “continuous landscape” vs. “fragmented landscape” study design, where paired landscapes differ in both habitat amount and fragmentation (e.g., Dixo and Metzger 2009; Falk et al. 2011; Gonçalves-Souza et al. 2025; Fig. 1a). This design uses a space-for-time substitution, where continuous and fragmented landscapes are meant to represent a given landscape before and after removal of habitat, respectively. It is not possible to determine whether variation in the ecological response between such continuous and fragmented landscapes is due to the difference in habitat amount, fragmentation, or their combination. Nevertheless, when ecological responses are lower in fragmented than continuous landscapes (e.g., fewer species in the fragmented landscapes), researchers have generally concluded that both habitat loss and fragmentation play a role in the responses—i.e., both have negative effects (e.g., Newmark and Stanley 2011; Capizzi et al. 2015; Botsch et al. 2017).

Researchers who find neutral or positive fragmentation effects typically compare ecological responses in landscapes meant to represent alternative endpoints of a given level of habitat loss (Fig. 1b), or they control statistically for habitat amount effects (fragmentation per se, sensu Haila and Hanski 1984; Fahrig 2003). Studies that estimate the independent effects of habitat loss and fragmentation through study design (e.g., Ethier and Fahrig 2011; Yan et al. 2024) and/or statistical approaches (e.g., Smith et al. 2011) find that effects of habitat loss are generally much stronger than effects of habitat fragmentation (reviewed in Fahrig 2003). Therefore, in studies that combine habitat loss and fragmentation, the strong and negative effects of habitat loss are likely masking weak—and often positive—effects of fragmentation.

**Fig. 2:**
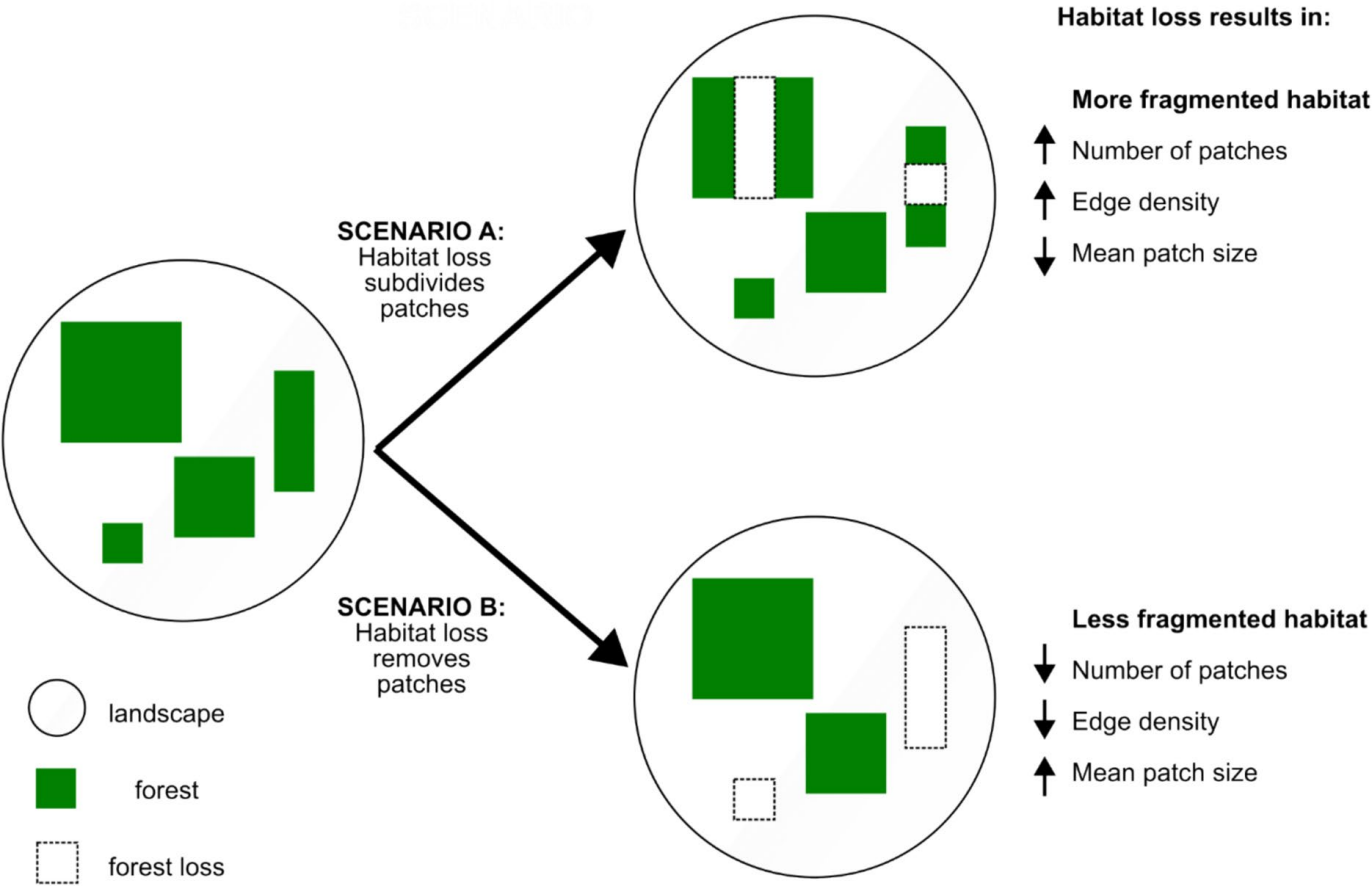
Illustrative examples to demonstrate that habitat loss could, in principle, result in an increase or decrease in fragmentation, depending on how habitat is removed. In scenario A, habitat becomes more fragmented due to habitat loss, with an increase in the number of patches, increase in edge density, and decrease in mean patch size. In contrast, scenario B has less fragmented habitat after habitat loss, i.e., fewer patches, lower edge density, and a larger mean patch size

The conceptualization and study of habitat fragmentation as a combination of habitat amount and fragmentation is rooted in the idea that habitat loss and habitat fragmentation are inherently linked, primarily because the breaking apart of habitat into more, smaller patches cannot occur without habitat loss (e.g., Ewers and Didham 2007; Didham et al. 2012; Fletcher et al. 2018; Banks-Leite et al. 2020). In contrast, a common argument for focusing on fragmentation per se is that, although habitat fragmentation results from habitat loss, habitat loss does not necessarily result in fragmentation (e.g., Fahrig 2003, 2018; Martin et al. 2021; Riva et al. 2024). For example, habitat loss can decrease fragmentation if a whole patch is removed from the landscape, thus reducing the number of patches in the landscape. While a decrease in habitat fragmentation with loss can be easily depicted in illustrative examples—including Fig. 2—it is possible that in the real world, human-caused habitat loss almost always leads to increased fragmentation. If true, we would expect that, in real landscapes, habitat loss and fragmentation are generally correlated through time such that habitat loss consistently results in increasing fragmentation. Studies of habitat fragmentation per se might be irrelevant to global biodiversity conservation if habitat loss and fragmentation are inevitably linked.

**Fig. 3:**
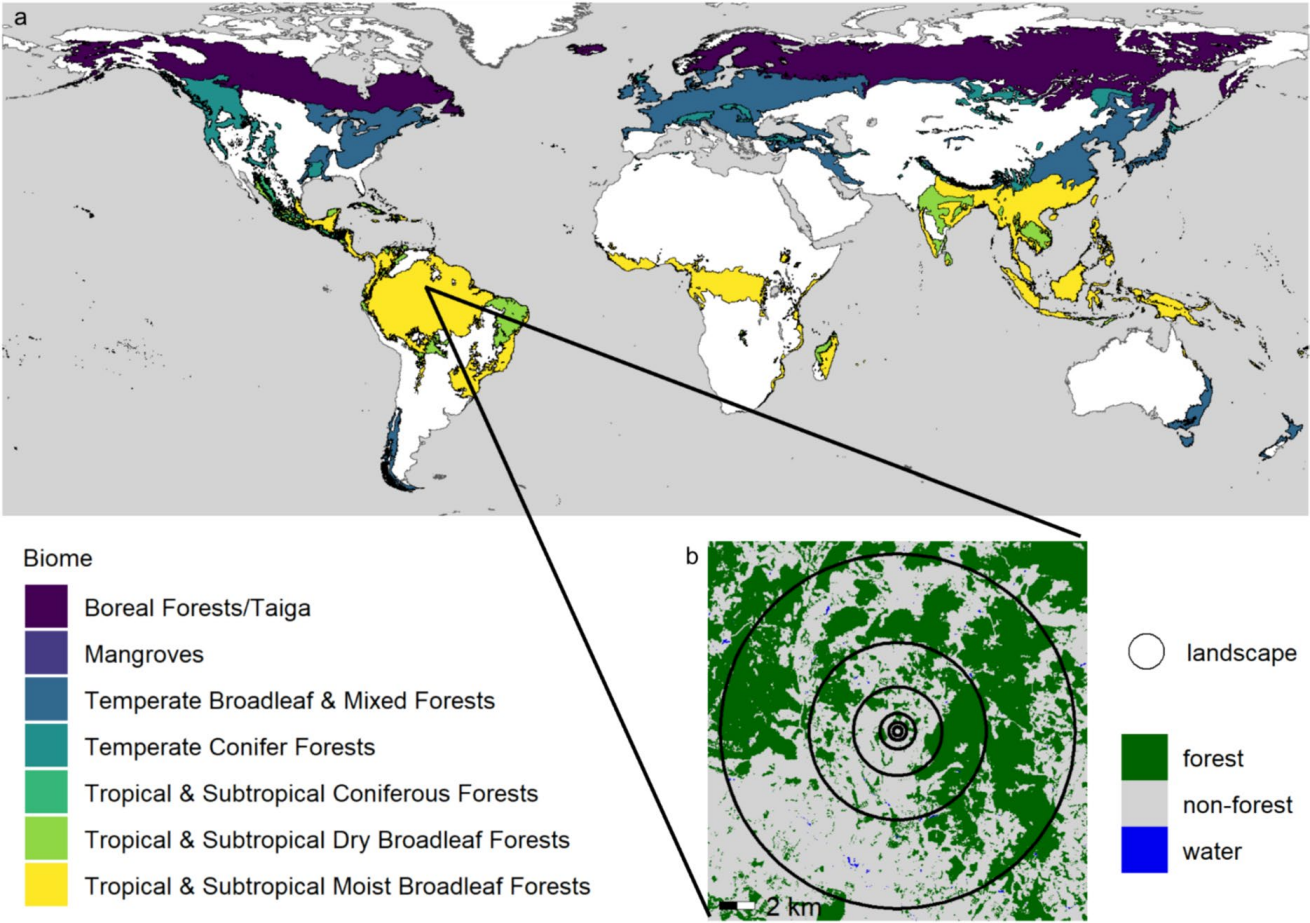
**a** The global distribution of forested biomes, as defined by Dinerstein et al. (2017). We sampled 150,000 random locations across these seven biomes. Around each sampled location, we measured the proportion of the landscape in forest, number of forest patches, edge density, and mean patch size. We evaluated landscapes of six sizes, delineated as circular buffers around the sampled location, with radii of 0.25, 0.5, 1, 2.5, 5, and 10 km. **b** Example sampling location. The four forest measurements were repeated twice for each location, once using forest cover data from 2000 and again using forest cover data from 2020. Forest cover data were derived from Potapov et al. (2022) and biomes are from Resolve (2017)

In this study, we evaluate how forest fragmentation changes as forest is lost, using three common measures of habitat fragmentation—number of patches, edge density, and mean patch size—and a fourth synthetic “forest fragmentation index” proposed by Ma et al. (2023). We do so for landscapes distributed across the world’s seven forested biomes (as defined by Dinerstein et al. 2017; Fig. 3a), using global maps of land use change from 2000 to 2020 (Potapov et al. 2022). To our knowledge, this is the first study to demonstrate that decreasing forest fragmentation is a frequent outcome of forest loss across a range of scales commonly used in landscape ecology studies.

## Methods

We selected a candidate set of 150,000 landscapes by randomly placing points across the seven forested biomes identified by Dinerstein et al. (2017; Fig. 3a). We expected this sample size would provide a reasonable representation of different patterns of forest loss and fragmentation occurring at the global scale; supplementary analysis confirmed that our results were consistent across a range of alternative sample sizes (Figs. S1–S4 in Online Resource 1). We then defined circular landscapes centered on each of the points. To evaluate the possibility that changes in fragmentation with forest loss are scale-dependent, we created six landscape sizes for each point (radii of 0.25, 0.5, 1, 2.5, 5, and 10 km; Fig. 3b). These scales were selected to represent a common range of landscape sizes considered in landscape ecology studies (as reviewed in Jackson and Fahrig 2015; Martin 2018; Arroyo-Rodríguez et al. 2023).

We derived global maps of forest cover in the years 2000 and 2020 from datasets created by Potapov et al. (2022) of forest extent and height in each year, plus the extents of cropland, built-up lands, surface water, snow, and ice, at a 30-m resolution. We created binary forest–non-forest maps for 2000 and 2020, classifying all areas with tree height ≥ 5 m as forest, consistent with the definition of forest used by the United Nations (FAO 2018). All other lands and waters were classified as non-forest.

For each sampled point, landscape size, and year of forest mapping, we projected the map to the Universal Transverse Mercator coordinate system, then calculated the: (1) proportion of the landscape in forest, (2) number of patches, (3) edge density (meters of forest–non-forest edge per ha), and (4) mean patch size (ha). For the subset of landscapes that lost forest between 2000 and 2020, we also calculated the forest fragmentation index using methods detailed in Ma et al. (2023), separately for each landscape size. This index was calculated in three steps. First, thresholds for extreme values of patch number, edge density, and mean patch size were identified, using the following formulas and forest measurements from the year 2020:

$${\mathrm{Q}}_{{{\mathrm{lower}}}} = {\mathrm{Q}}_{{1}} + {1}.{5} \times {\mathrm{IQR}}$$

$${\mathrm{Q}}_{{{\mathrm{upper}}}} = {\mathrm{Q}}_{{3}} + {1}.{5} \times {\mathrm{IQR}}$$

where Q_lower_ and Q_upper_ represent thresholds for extreme low and high values, respectively; Q1 = first quartile, Q3 = third quartile, and IQR = interquartile range (Q3–Q1). Values below and above Q_lower_ and Q_upper_, respectively, were set to the values of Q_lower_ and Q_upper_. These same Q_lower_ and Q_upper_ values were used to adjust extreme values in the year 2000. Second, patch number, edge density, and mean patch size were normalized to a range of zero to one in each year. Finally, the forest fragmentation index was calculated as:

Forest fragmentation index = (PN + ED + [1 – MPA]) / 3

where PN = normalized patch number for a sampled point, landscape size, and year, ED = normalized edge density, and MPA = normalized mean patch area. Ma et al. (2023) collapsed values below and above Q_lower_ and Q_upper_, respectively, to limit influence of outliers on their measure of fragmentation. We assessed whether our conclusions would change if we used a modified version of this forest fragmentation index that included the full range of patch numbers, edge densities, and mean patch sizes. Our overall conclusions did not change (Figs. S1–S3 in Online Resource 2).

We calculated measures of forest loss and changes in fragmentation for retained landscapes, separately for each landscape size. Forest loss was the proportion of the landscape in forest in 2000 minus the proportion in 2020. Thus, larger values indicate greater net loss of forest. For each of the four measures of fragmentation, we measured its change as the value in 2020 minus the value in 2000. Positive changes in patch number, edge density, and the forest fragmentation index indicate increases in fragmentation, i.e., more patches, more edge, and a higher overall index value in 2020 than in 2000. For mean patch size, positive values indicate decreasing fragmentation, i.e., larger average patch sizes in 2020 than in 2000. Therefore, we present the reversed values for changes in mean patch size in our figures (− 1 × change in mean patch size), so that positive values indicate increasing fragmentation across all four measures.

Finally, we assessed the percentage of landscapes with increasing, decreasing, or no change in forest fragmentation, separately for each measure of fragmentation and landscape size. We also investigated whether increases/decreases in fragmentation varied with the (1) proportion of the original (i.e., in 2000) landscape in forest, (2) the proportion of forest lost from 2000 to 2020, and (3) biome. See Figs. S1–S3 in Online Resource 3 for plots of relationships among these three variables.

## Results

The percentage of landscapes that lost forest from 2000 to 2020 (rather than gaining forest or having no change in forest cover) depended on the landscape size, increasing from 29.8% of landscapes at the 0.25-km scale to 57.0% of landscapes at the 10-km scale (Fig. 1 in Online Resource 4). Landscapes that lost forest tended to have high forest cover in 2000 (Table S1, Fig. S2 in Online Resource 4). Average proportions of forest lost over the two-decade period ranged from 0.11 to 0.04, from the smallest to the largest landscape size, respectively (Table S1, Fig. S3 in Online Resource 4).

**Fig. 4:**
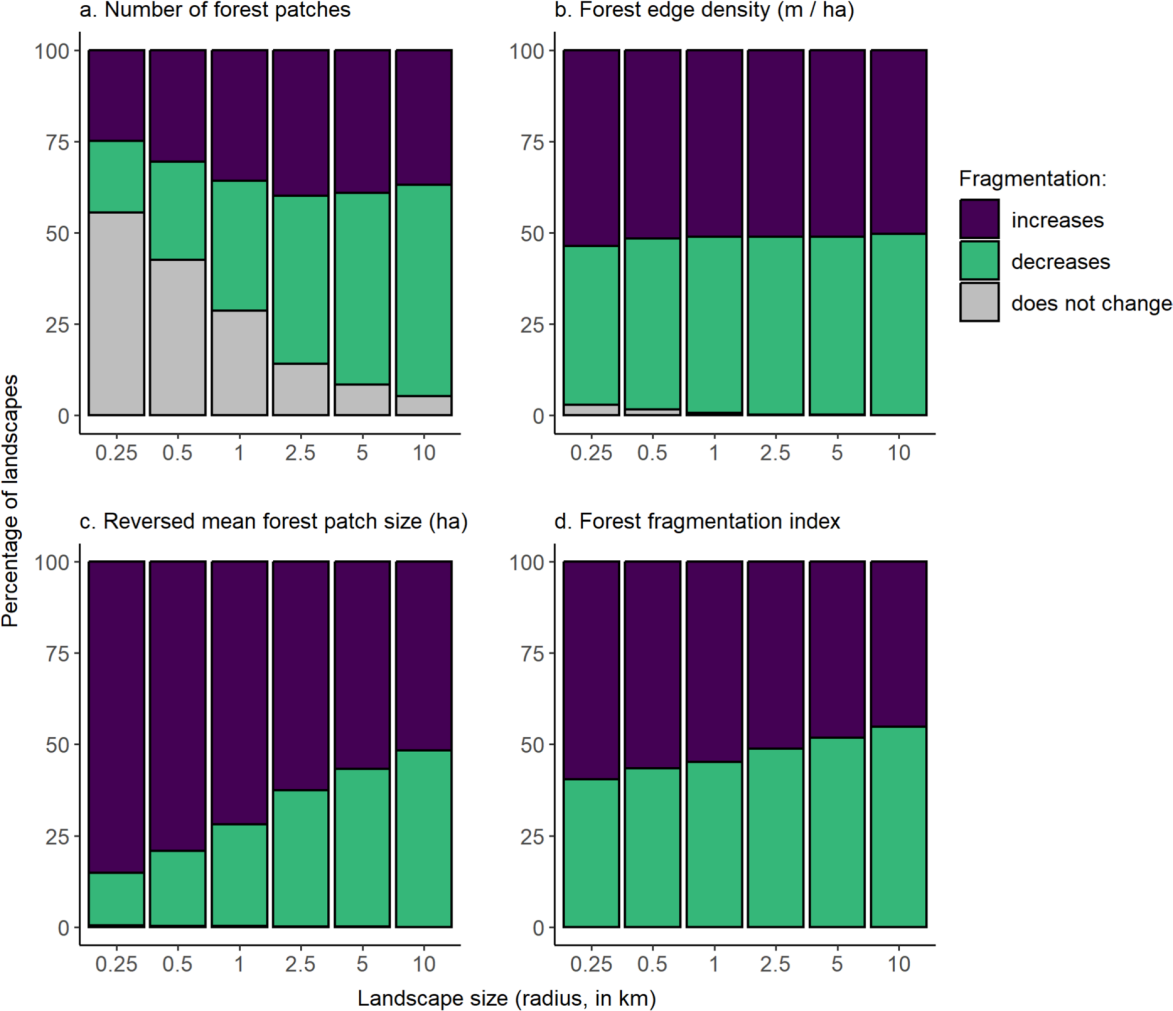
Percentages of landscapes with forests that became more fragmented, less fragmented, and showed no change in fragmentation after forest loss, for each of six landscape sizes and four measures of fragmentation. Fragmentation was classified as increasing when there were **a** more forest patches, **b** higher forest edge density (meters of forest–non-forest edge per ha, including all forest patches in the landscape), **c** smaller mean forest patch sizes, and **d** higher forest fragmentation index values in 2020 than in 2000. The total number of landscapes ranged from 36,482 at the smallest landscape size to 84,635 at the largest landscape size (Table S1 in Online Resource 4); only landscapes that lost forest between 2000 and 2020 are included

A decrease in fragmentation was a common outcome of forest loss. Forest loss caused decreases in the number of patches almost as often as it caused increases (Fig. 4a). There were decreases in the number of patches in 20–58% of landscapes for the smallest to largest landscape sizes. Patch number increased in 25–37% of landscapes, for the smallest to largest landscape sizes. Edge density declined in approximately half of landscapes after forest loss (44–50% of landscapes, depending on the landscape size; Fig. 4b). Decreases and increases in mean forest patch size in response to forest loss occurred with similar frequencies in large landscapes, with increases in mean patch size (decreased fragmentation) in 48% of the largest landscapes (Fig. 4c). However, in smaller landscapes forest loss typically resulted in reduced mean patch size (increased fragmentation); at the smallest landscape size, reduced mean patch size was observed in 85% of landscapes (Fig. 4c). Using the synthetic forest fragmentation index, we found that the percentage of landscapes showing a decrease in fragmentation with forest loss ranged from 40 to 55%, from the smallest to largest landscapes size (Fig. 4d).

**Fig. 5:**
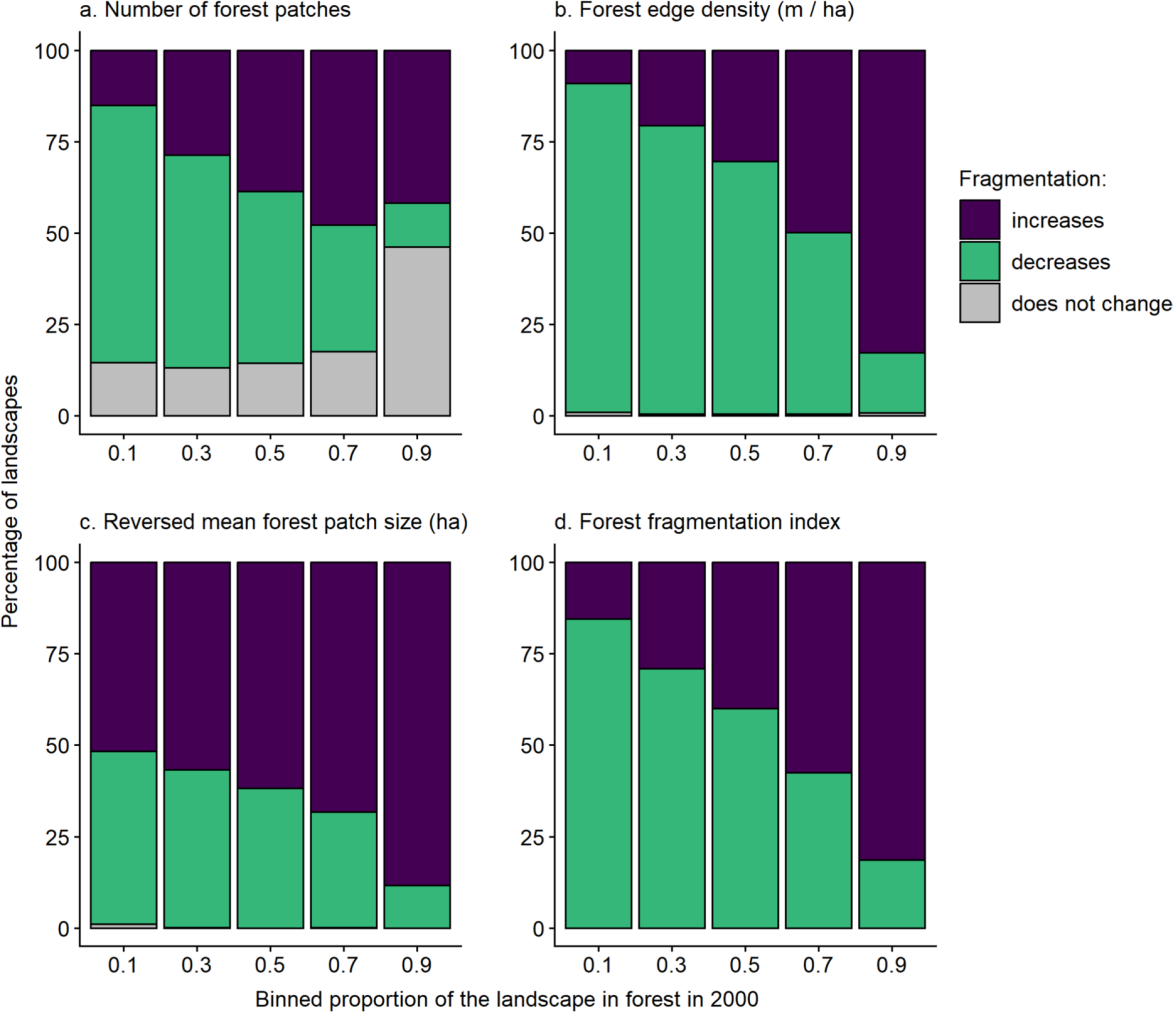
Relationships between the proportion of the landscape in forest in 2000 and the percentages of landscapes that became more fragmented, less fragmented, and showed no change in fragmentation associated with forest loss. The proportion of the landscape in forest was binned into 0.2 intervals. Fragmentation was classified as increasing when there were **a** more forest patches, **b** higher forest edge density (meters of forest–non-forest edge per ha, including all forest patches in the landscape), **c** smaller mean forest patch sizes, and **d** higher forest fragmentation index values in 2020 than in 2000. Results are for landscapes with a 1-km radius (n = 63,019); only landscapes that lost forest between 2000 and 2020 are included

**Fig. 6:**
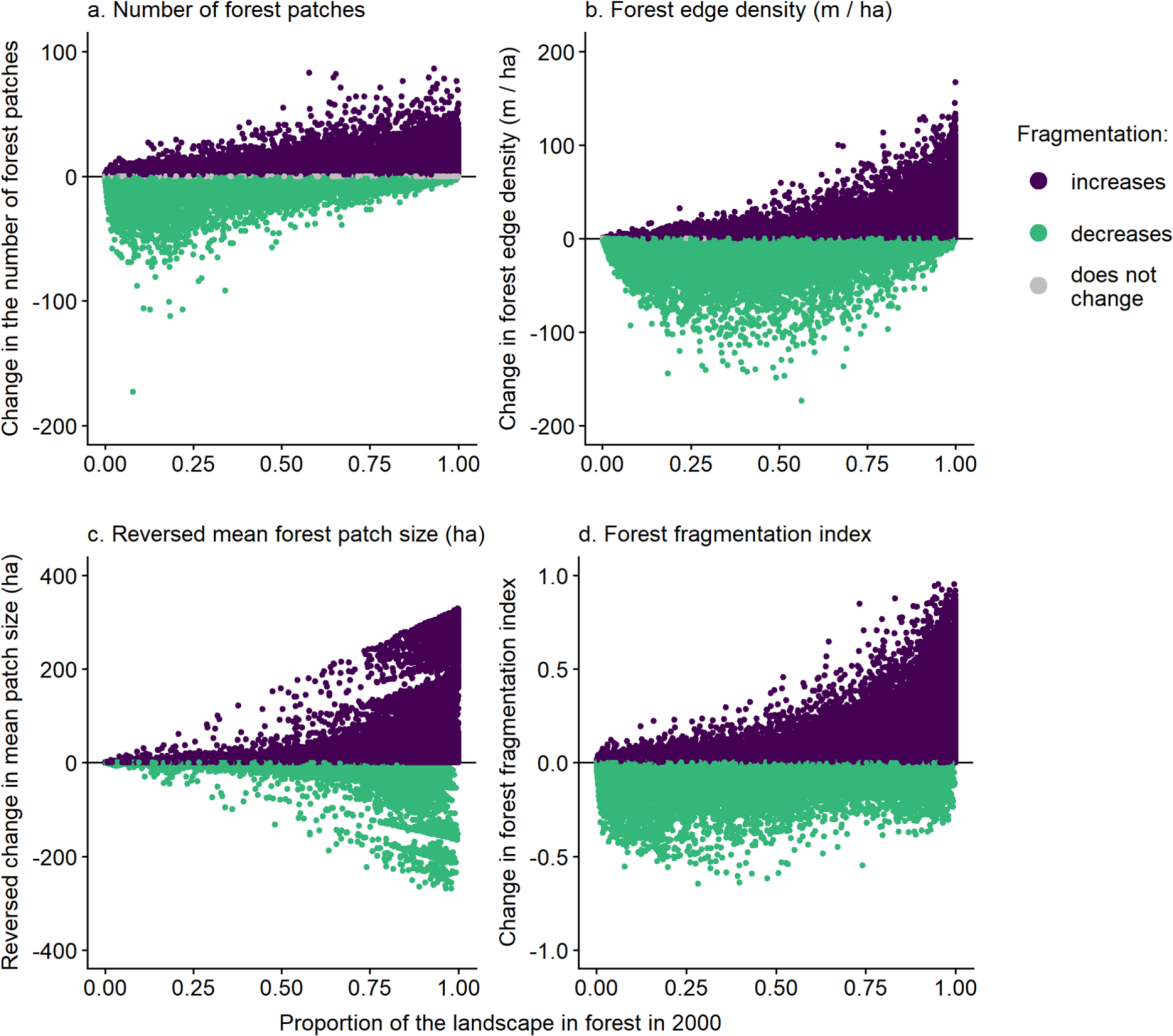
Relationships between the proportion of the landscape in forest in 2000 and the change in forest fragmentation from 2000 to 2020, for each of four measures of forest fragmentation. Each point is a landscape. An increase in fragmentation is indicated by **a** more forest patches, **b** higher forest edge density (meters of forest–non-forest edge per ha, including all forest patches in the landscape), **c** smaller mean forest patch sizes, and **d** higher forest fragmentation index values in 2020 than in 2000. We plot the reversed change in mean patch size, so that increased fragmentation is associated with positive values across all measures. Results are for 1-km-radius landscapes (n = 63,019); only landscapes that lost forest between 2000 and 2020 are included

Whether forest loss caused a decrease or increase in fragmentation varied with the amount of forest in the landscape, the biome, and the magnitude of forest loss. Patterns were similar for all landscape sizes; thus, for simplicity, we focus on the 1-km-radius landscape size here (see Figs. S1–S10 in Online Resource 5 for results using the smallest and largest landscape sizes). Forest loss tended to decrease fragmentation when habitat was removed from landscapes with low forest amounts, and the opposite in landscapes with high forest amounts. This pattern was consistent across all indices of fragmentation (Figs. 5, 6). However, for mean patch size the largest increases and decreases in fragmentation occurred in landscapes with the most forest (Fig. 6c). Forest loss was most likely to increase fragmentation in Temperate Conifer Forests and most likely to decrease fragmentation in Tropical & Subtropical Dry Broadleaf Forests (Fig. S4 in Online Resource 4). Finally, increasing fragmentation was a more common outcome than decreasing fragmentation in landscapes that lost a larger amount of forest cover (Figs. S5, S6 in Online Resource 4). However, losses of large amounts of forest were uncommon, e.g., only 6% of landscapes lost > 0.30 of their forest between 2000 and 2020.

## Discussion

Our global-scale analysis shows that forest loss commonly results in both increases and decreases in forest fragmentation. The number of landscapes showing decreasing versus increasing fragmentation after forest loss depended on both the fragmentation measure and spatial scale of its measurement. However, across all four indices of fragmentation, six landscape sizes, and all forested biomes, we found thousands of cases of landscapes where forest loss led to lower fragmentation. In initiating this study, we realized that either outcome was theoretically possible (Fig. 2). However, it is important to seek empirical support for theoretical expectations. Our findings show that habitat loss also commonly reduces habitat fragmentation in real landscapes.

Two other recent, global-scale studies evaluated changes in fragmentation over time in all forest types (Ma et al. 2023; Woodman et al. 2025). Our analyses differ from these previous studies in that we assessed a range of spatial scales representative of scales commonly used in landscape ecology studies (as reviewed in Jackson and Fahrig 2015; Martin 2018; Arroyo-Rodríguez et al. 2023). That is, we assessed landscapes ranging from 0.2 to 314 km^2^, whereas Ma et al. (2023) and Woodman et al. (2025) used areas of 25 km^2^ and 100–25,6000 km^2^, respectively. Our objective was also different, focusing specifically on the question of whether it is reasonable to expect decreasing fragmentation following forest loss in real landscapes. Despite differences in spatial scale and intent of study, our results are generally consistent with those of Ma et al. (2023) and Woodman et al. (2025). Ma et al. (2023) summarized dominant patterns of forest amount and fragmentation change by country, and reported that, when countries predominantly lost forest, their fragmentation was slightly more likely to decrease than increase (in 54 versus 32 countries, respectively). Woodman et al. (2025) also found that increases and decreases in fragmentation occurred across the subset of landscapes that lost forest, although in this case increasing fragmentation was a more likely outcome than decreasing fragmentation.

We recognize that land cover classifications from remotely-sensed imagery—such as what we (and Ma et al. 2023 and Woodman et al. 2025) used—are subject to uncertainty, particularly when the objects mapped (trees) are smaller than the resolution of the imagery. This can, for example, lead to classification uncertainty at boundaries between forest and other land cover classes. In turn, classification uncertainty could cause spurious detection of changes in forest cover and fragmentation between 2000 and 2020, if that uncertainty leads to pixels at forest edges being erroneously classified as forest in one year and correctly classified as non-forest in the other year. It is also possible that classification of single, isolated pixels as forest (or small groups of pixels as forest) may be more likely to reflect classification errors than larger, contiguous groups of pixels. This could, for example, cause spurious detection of changes in forest cover and fragmentation if the isolated pixel is erroneously classified as forest in one year and correctly classified as non-forest in the other year. We investigated whether these two issues could influence our conclusions. They did not. Across all sensitivity analyses we found thousands of cases where forest loss resulted in decreasing fragmentation (Figs. S1–S3 in Online Resource 6).

Whether studying habitat fragmentation independent from habitat loss (i.e., fragmentation per se) is meaningful for biodiversity conservation depends on whether habitat fragmentation is a (nearly) inevitable consequence of habitat loss. The evidence here suggests that fragmentation can increase or decrease with habitat loss. It is therefore meaningful to evaluate the relative effects of habitat amount and fragmentation, and particularly how fragmentation affects biodiversity independent of habitat amount, to inform global biodiversity conservation.

Two key conservation recommendations can be derived from a synthesis of the current literature on the effects of fragmentation per se. First, conservation policy and action should focus on protecting and restoring natural habitat, regardless of how that habitat is arranged in the landscape. Studies of fragmentation per se have shown that habitat loss has negative effects on biodiversity that are typically much larger than the effects of the resulting change in habitat pattern (Fahrig 2003). Thus, attempts to alter the level of fragmentation resulting from a given loss of habitat generally will not compensate for the negative effects of habitat loss on biodiversity. Second, policymakers and conservation practitioners should value maintaining small habitat remnants in human-modified landscapes. Evidence suggests that effects of fragmentation per se are usually neutral or slightly positive (Fahrig 2003, 2017). This means that a given area of habitat in small patches is of equal or more value for biodiversity conservation to the same area in one or a few large patches. This conclusion is particularly important because studies that confound effects of habitat loss and fragmentation (e.g., Gonçalves-Souza et al. 2025; Fahrig et al. 2026) have likely contributed to the perception that small habitat patches have low value for biodiversity conservation, despite evidence to the contrary (e.g., Bennett and Arcese 2013; Tulloch et al. 2016; Wintle et al. 2019; Riva and Fahrig 2022). Indeed, some conservation policies/guidelines specifically favor retention of large forest patches (see Riva et al. 2024 and references therein). A preference for larger over smaller patches seems to be reflected in global forest loss trends; for example, Riva et al. (2022) found that a given area of forest is more likely to be lost from a small patch than a large one. This devaluation of small patches impacts biodiversity especially in regions where most of the remaining habitat is in small patches. For example, ~ 50% of landscapes in this study had a mean forest patch size of < 20 ha in 2020 (Fig. S1 in Online Resource 7).

In conclusion, our results suggest that habitat fragmentation can and should be studied independently of habitat amount. The fact that habitat fragmentation results from habitat loss does not mean that fragmentation is the inevitable consequence of habitat loss. In fact, across the Earth's forests, forest loss is increasing and decreasing fragmentation with about equal likelihood. Understanding the effects of fragmentation independent of the effects of habitat loss is not only feasible, it is also important for developing effective habitat conservation plans to address the ongoing crisis of biodiversity loss.

## Supplementary Information

Below is the link to the electronic supplementary material.

Supplementary file1 (DOCX 290 KB)

Supplementary file2 (DOCX 167 KB)

Supplementary file3 (DOCX 249 KB)

Supplementary file4 (DOCX 336 KB)

Supplementary file5 (DOCX 698 KB)

Supplementary file6 (DOCX 385 KB)

Supplementary file7 (DOCX 40 KB)

## Data Availability

Landcover data are publicly available from [https://glad.earthengine.app/view/glcluc-2000-2020] (https://glad.earthengine.app/view/glcluc-2000-2020) (Potapov et al. 2022). Data used to delineate forested biomes are publicly available from [https://ecoregions.appspot.com/] (https://ecoregions.appspot.com) (Resolve 2017). All other data and R code are available from 10.6084/m9.figshare.29987362 (Martin et al. 2026).

## References

[CR1] Arroyo-Rodríguez V, Martínez-Ruiz M, Bezerra JS et al (2023) Does a species’ mobility determine the scale at which it is influenced by the surrounding landscape pattern? Curr Landsc Ecol Rep 8:23–33

[CR2] Banks-Leite C, Ewers RM, Folkard-Tapp H, Fraser A (2020) Countering the effects of habitat loss, fragmentation, and degradation through habitat restoration. One Earth 3:672–676

[CR3] Bennett JR, Arcese P (2013) Human influence and classical biogeographic predictors of rare species occurrence. Conserv Biol 27:417–421 23330728 10.1111/cobi.12015

[CR4] Botsch JC, Walter ST, Karubian J et al (2017) Impacts of forest fragmentation on orchid bee (Hymenoptera: Apidae: Euglossini) communities in the Chocó biodiversity hotspot of northwest Ecuador. J Insect Conserv 21:633–643

[CR5] Capizzi D, Luiselli L, Papi R (2015) Temporal changes in Mediterranean bird communities across fragmented and continuous forests. Ecol Res 30:615–624

[CR6] Didham RK, Kapos V, Ewers RM (2012) Rethinking the conceptual foundations of habitat fragmentation research. Oikos 121:161–170

[CR7] Dinerstein E, Olson D, Joshi A et al (2017) An ecoregion-based approach to protecting half the terrestrial realm. Bioscience 67:534–545 28608869 10.1093/biosci/bix014 PMC5451287

[CR8] Dixo M, Metzger JP (2009) Are corridors, fragment size and forest structure important for the conservation of leaf-litter lizards in a fragmented landscape? Oryx 43:435–442

[CR9] Ethier K, Fahrig L (2011) Positive effects of forest fragmentation, independent of forest amount, on bat abundance in eastern Ontario, Canada. Landsc Ecol 26:865–876

[CR10] Ewers RM, Didham RK (2007) Habitat fragmentation: panchreston or paradigm? Trends Ecol Evol 22:P511 17624622 10.1016/j.tree.2007.06.004

[CR12] Fahrig L (2003) Effects of habitat fragmentation on biodiversity. Annu Rev Ecol Evol Syst 34:487–515

[CR11] Fahrig L (2017) Ecological responses to habitat fragmentation per se. Annu Rev Ecol Evol Syst 48:1–23

[CR13] Fahrig L (2018) Forty years of bias in habitat fragmentation research. In: Kareiva P, Marvier M, Silliman B (eds) Effective conservation science: data not dogma. Oxford University Press, Oxford, pp 32–38

[CR14] Fahrig L, Arroyo-Rodríguez V, Bennett JR et al (2019) Is habitat fragmentation bad for biodiversity? Biol Conserv 230:179–186

[CR15] Fahrig L, Galán-Acedo C, Edward BPM et al (2026) Why controlling for habitat amount is critical for resolving the fragmentation debate. Conserv Biol 2026:e70245 10.1111/cobi.70245 41684085

[CR16] Falk KJ, Nol E, Burke DM (2011) Weak effect of edges on avian nesting success in fragmented and forested landscapes in Ontario, Canada. Landsc Ecol 26:239–251

[CR17] FAO (2018) Terms and definitions: FRA 2020. Food and Agriculture Organization of the United Nations, Rome

[CR18] Fletcher RJ, Didham RK, Banks-Leite C et al (2018) Is habitat fragmentation good for biodiversity? Biol Conserv 226:9–15

[CR19] Gonçalves-Souza T, Chase JM, Haddad NM et al (2025) Species turnover does not rescue biodiversity in fragmented landscapes. Nature 640:702–706 40074894 10.1038/s41586-025-08688-7

[CR20] Haddad NM, Brudvig LA, Clobert J et al (2015) Habitat fragmentation and its lasting impact on Earth’s ecosystems. Sci Adv 1:e1500052 26601154 10.1126/sciadv.1500052 PMC4643828

[CR21] Haila Y, Hanski IK (1984) Methodology for studying the effect of habitat fragmentation on land birds. Ann Zool Fenn 21:393–397

[CR22] Jackson HB, Fahrig L (2015) Are ecologists conducting research at the optimal scale? Glob Ecol Biogeogr 24:52–63

[CR23] Ma J, Li J, Wu W, Liu J (2023) Global forest fragmentation change from 2000 to 2020. Nat Commun 14:3752 37433782 10.1038/s41467-023-39221-x PMC10336092

[CR24] Martin AE (2018) The spatial scale of a species’ response to the landscape context depends on which biological response you measure. Curr Landsc Ecol Rep 3:23–33

[CR25] Martin AE, Bennett JR, Fahrig L (2021) Influence of habitat loss and fragmentation on wildlife populations. In: Porter WF, Parent CJ, Stewart RA, Williams DM (eds) Wildlife management and landscapes: principles and applications. Johns Hopkins University Press, Baltimore, pp 96–113

[CR39] Martin A, Galán-Acedo C, Riva F (2026) Fahrig L Data and code from: Habitat fragmentation can either increase or decrease with habitat loss, *Figshare*, 10.6084/m9.figshare.29987362 10.1007/s10980-026-02345-8 PMC13194208 42179760

[CR26] Miller-Rushing AJ, Primack RB, Devictor V et al (2019) How does habitat fragmentation affect biodiversity? A controversial question at the core of conservation biology. Biol Conserv 232:271–273

[CR27] Newmark WD, Stanley TR (2011) Habitat fragmentation reduces nest survival in an Afrotropical bird community in a biodiversity hotspot. Proc Natl Acad Sci USA 108:11488–11493 21709237 10.1073/pnas.1104955108 PMC3136281

[CR28] Potapov P, Hansen MC, Pickens A et al (2022) The global 2000-2020 land cover and land use change dataset derived from the Landsat archive: first results. Front Remote Sens 3:856903

[CR29] Resolve (2017) Ecoregions 2017. https://ecoregions.appspot.com/ Accessed 4 Aug 2023.

[CR30] Riva F, Fahrig L (2022) The disproportionately high value of small patches for biodiversity conservation. Conserv Lett 15:e12881

[CR32] Riva F, Martin CJ, Millard K, Fahrig L (2022) Loss of the world’s smallest forests. Glob Chang Biol 28:7164–7166 36189962 10.1111/gcb.16449

[CR31] Riva F, Koper N, Fahrig L (2024) Overcoming confusion and stigma in habitat fragmentation research. Biol Rev 99:1411–1424 38477434 10.1111/brv.13073

[CR33] Smith AC, Fahrig L, Francis CM (2011) Landscape size affects the relative importance of habitat amount, habitat fragmentation, and matrix quality on forest birds. Ecography 34:103–113

[CR34] Tulloch AIT, Barnes MD, Ringma J et al (2016) Understanding the importance of small patches of habitat for conservation. J Appl Ecol 53:418–429

[CR35] Valente JJ, Gannon DG, Hightower J et al (2023) Toward conciliation in the habitat fragmentation and biodiversity debate. Landsc Ecol 38:2717–2730

[CR36] Wintle BA, Kujala H, Whitehead A et al (2019) Global synthesis of conservation studies reveals the importance of small habitat patches for biodiversity. Proc Natl Acad Sci USA 116:909–914 30530660 10.1073/pnas.1813051115 PMC6338828

[CR37] Woodman TL, Alexander P, Burslem DFRP et al (2025) Global assessment of landscape pattern changes from 1992 to 2020. Landsc Ecol 40:196. 10.1007/s10980-025-02210-0 PMC12532760 41113106

[CR38] Yan Y, Jarvie S, Zhang Q (2024) Habitat loss weakens the positive relationship between grassland plant richness and above-ground biomass. eLife 12:91193 38497752 10.7554/eLife.91193 PMC10948147

