# Supplementary material for: Habitat fragmentation can either increase or decrease with habitat loss
Source: Landsc Ecol. 2026 Apr 9;41(6):97. doi: 10.1007/s10980-026-02345-8 (PMC13194208; doi:10.1007/s10980-026-02345-8)
Supplement: Supplementary file 1 — Supplementary file1 (DOCX 290 KB) [file 10980_2026_2345_MOESM1_ESM.docx]

**Online Resource 1**


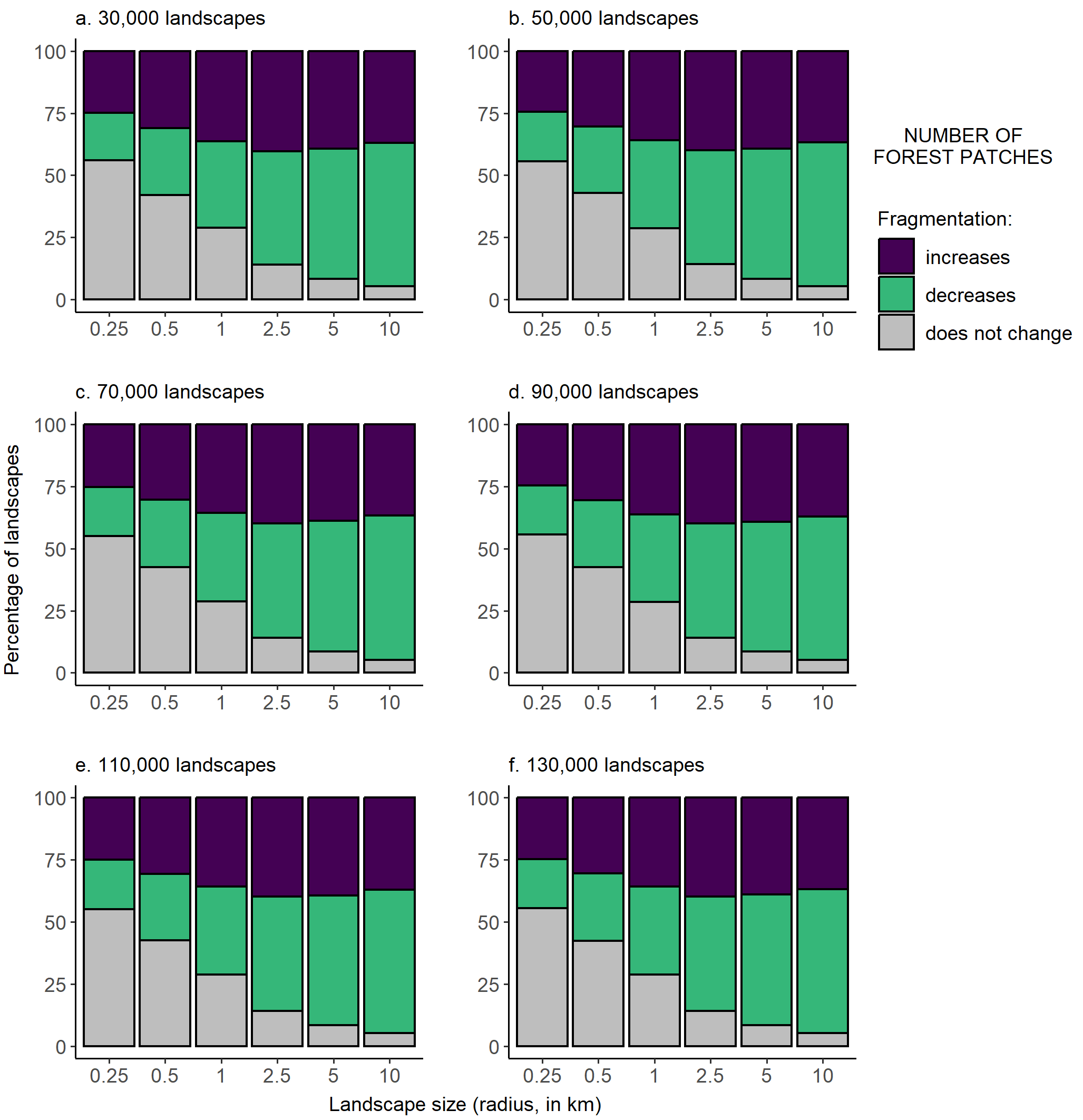


**Fig. S1** Percentages of landscapes with forests that became more fragmented, less fragmented, and showed no change in fragmentation after forest loss, for each of six landscape sizes and six alternative sample sizes (from 30,000 to 130,000 landscapes). Fragmentation was classified as increasing when there were more forest patches in 2020 than in 2000. Only the subset of landscapes that lost forest between 2000 and 2020 are included in the plots


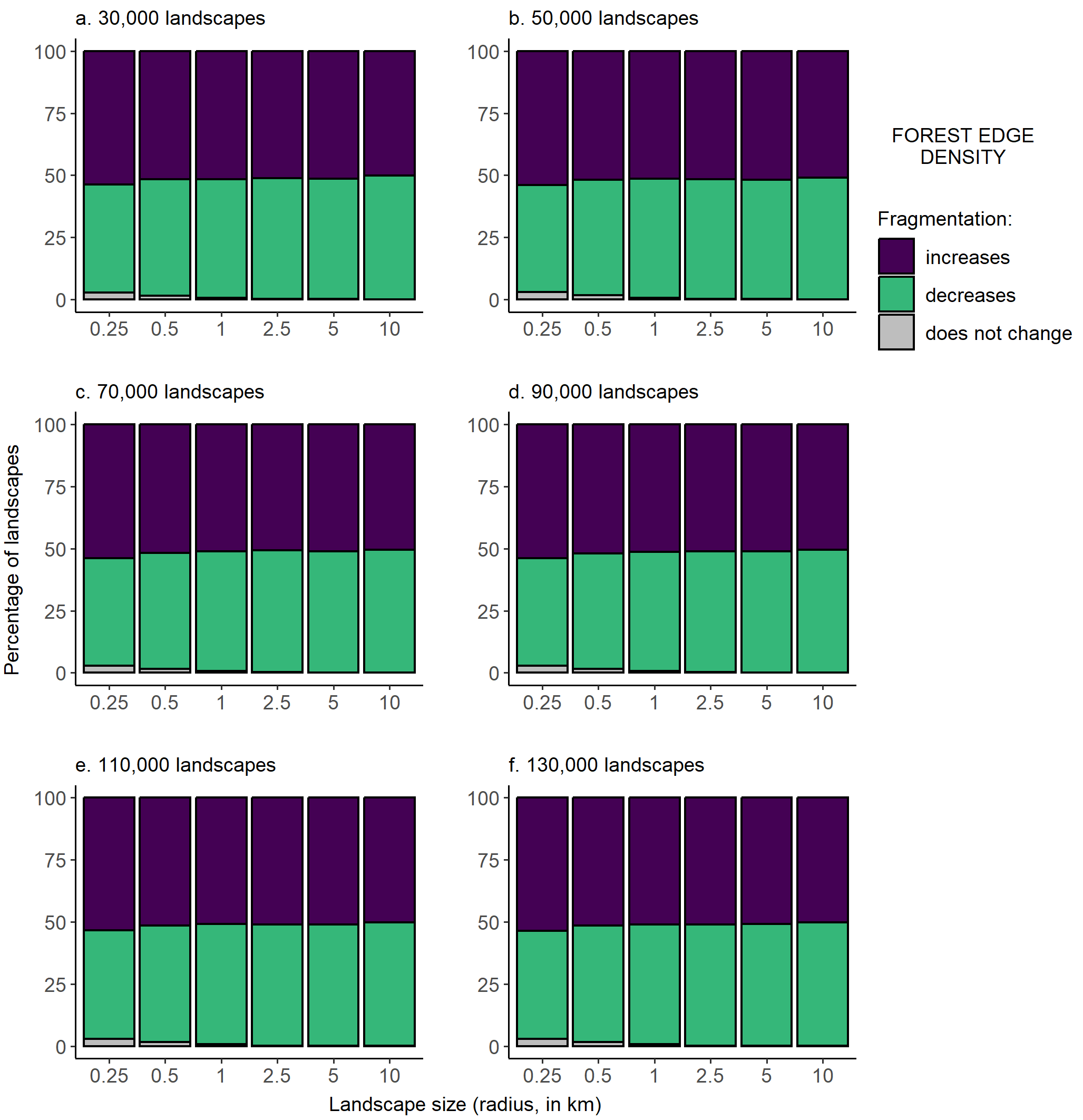


**Fig. S2** Percentages of landscapes with forests that became more fragmented, less fragmented, and showed no change in fragmentation after forest loss, for each of six landscape sizes and six alternative sample sizes (from 30,000 to 130,000 landscapes). Fragmentation was classified as increasing when there was higher forest edge density (meters of forest–non-forest edge per ha, including all patches in the landscape) in 2020 than in 2000. Only the subset of landscapes that lost forest between 2000 and 2020 are included in the plots


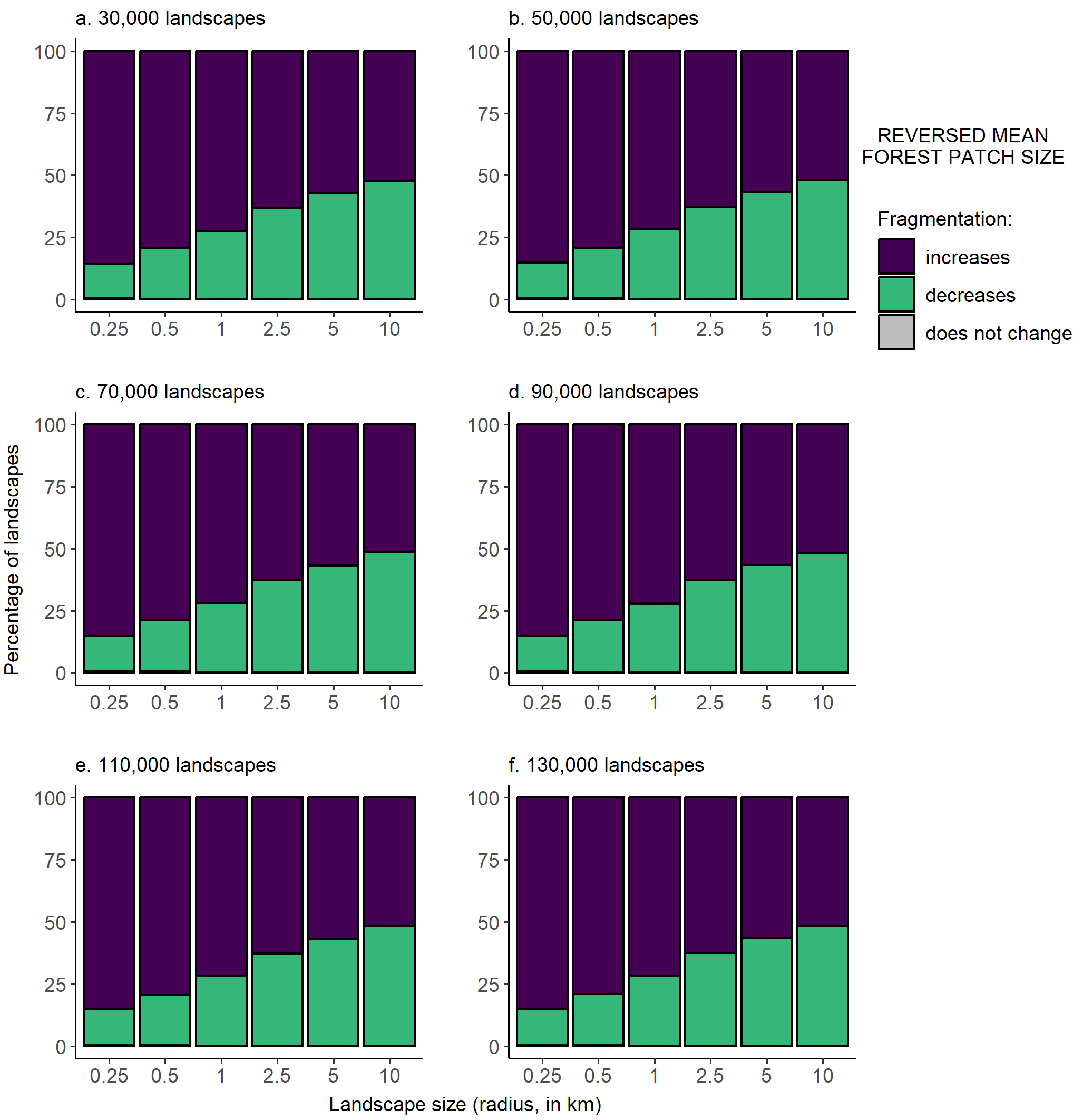


**Fig. S3** Percentages of landscapes with forests that became more fragmented, less fragmented, and showed no change in fragmentation after forest loss, for each of six landscape sizes and six alternative sample sizes (from 30,000 to 130,000 landscapes). Fragmentation was classified as increasing when there were smaller mean forest patch sizes in 2020 than in 2000. Only the subset of landscapes that lost forest between 2000 and 2020 are included in the plots


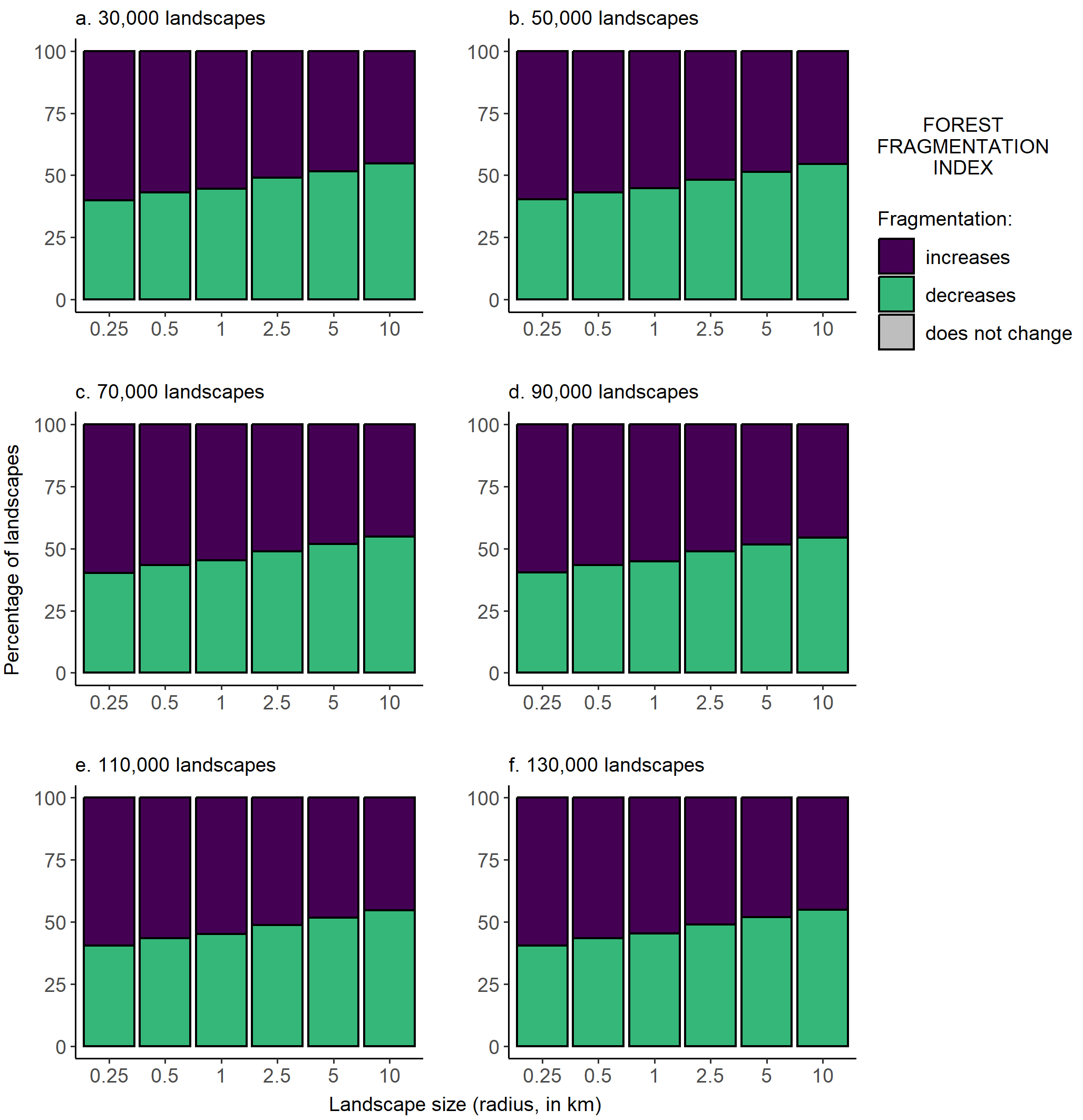


**Fig. S4** Percentages of landscapes with forests that became more fragmented, less fragmented, and showed no change in fragmentation after forest loss, for each of six landscape sizes and six alternative sample sizes (from 30,000 to 130,000 landscapes). Fragmentation was classified as increasing when there were higher forest fragmentation index values in 2020 than in 2000. Only the subset of landscapes that lost forest between 2000 and 2020 are included in the plots
