# Supplementary material for: Habitat fragmentation can either increase or decrease with habitat loss
Source: Landsc Ecol. 2026 Apr 9;41(6):97. doi: 10.1007/s10980-026-02345-8 (PMC13194208; doi:10.1007/s10980-026-02345-8)
Supplement: Supplementary file 2 — Supplementary file2 (DOCX 167 KB) [file 10980_2026_2345_MOESM2_ESM.docx]

**Online Resource 2**


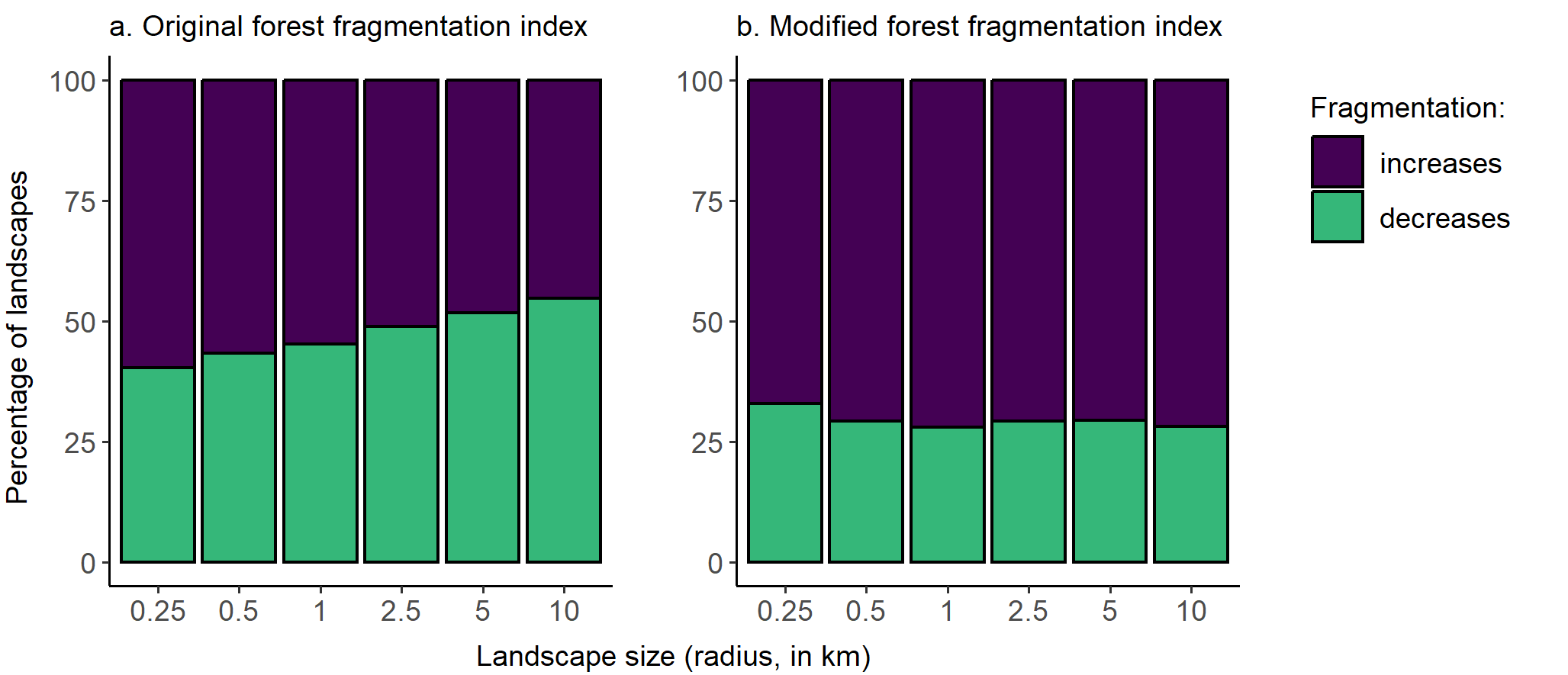


**Fig. S1** Comparison of the percentages of landscapes with forests that became more fragmented, less fragmented, and showed no change in fragmentation after forest loss, for each of six landscape sizes and two versions of the forest fragmentation index. (a) The original forest fragmentation index, calculated as described in Ma et al. (2023). (b) A modified forest fragmentation index, which included the full range of patch numbers, edge densities, and mean patch sizes rather than truncating outlier values. Fragmentation was classified as increasing when there were higher forest fragmentation index values in 2020 than in 2000. The total number of landscapes ranged from 36,482 at the smallest landscape size to 84,635 at the largest landscape size (Table S1 in Online Resource 4); only landscapes that lost forest between 2000 and 2020 are included. Although forest became more fragmented after forest loss more frequently when using the modified than original forest fragmentation index, our overall conclusions were the same. Across four indices of fragmentation (number of patches, edge density, mean forest patch size, and the forest fragmentation index), six landscape sizes, and all forested biomes, we found forests were less fragmented after forest loss 44% of the time when including the original forest fragmentation index and 39% of the time when using the modified forest fragmentation index


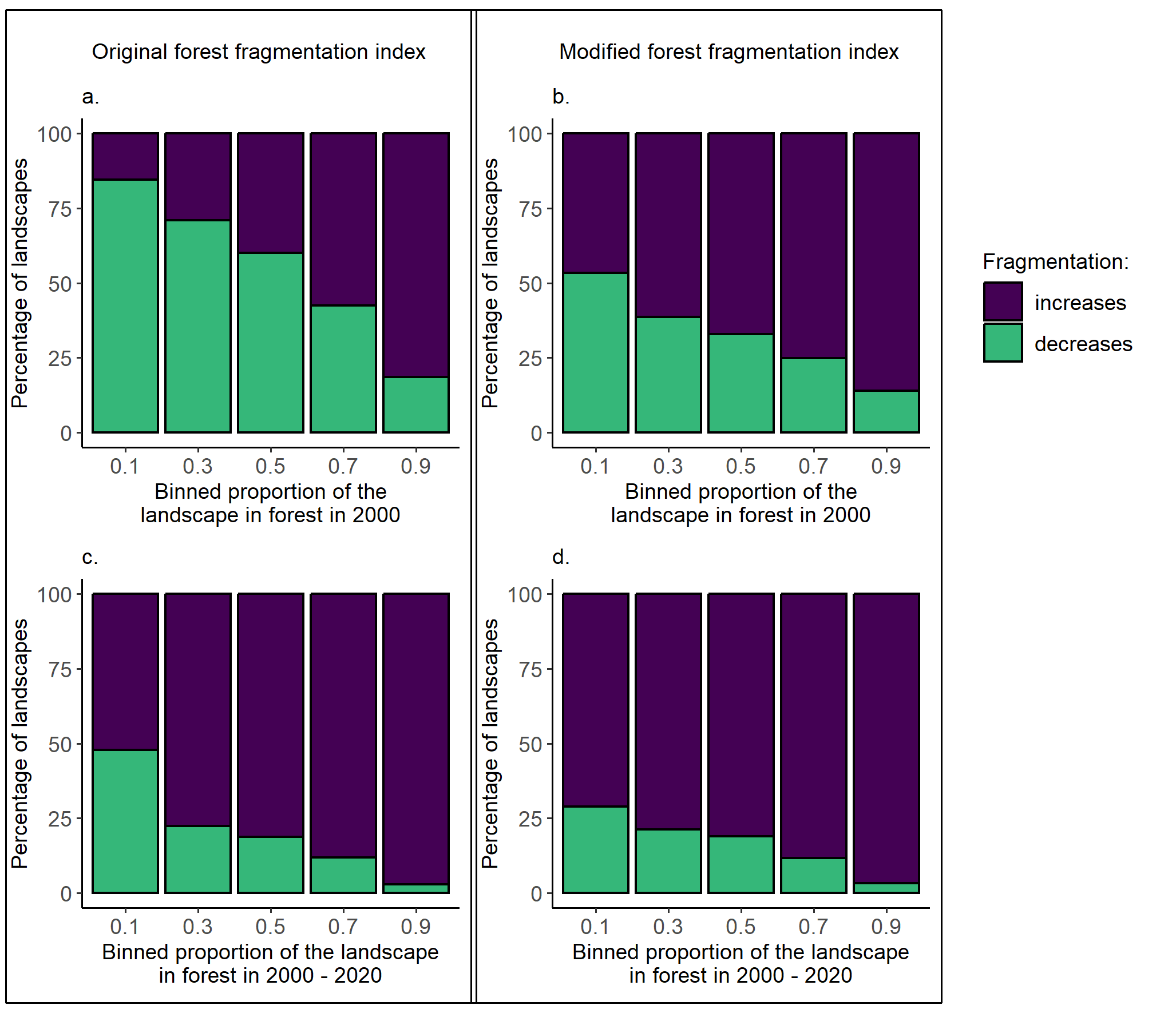


**Fig. S2** Relationships between the proportion of the landscape in forest in 2000 (a–b) or forest loss (c–d) and the percentages of landscapes that became more fragmented, less fragmented, and showed no change in fragmentation associated with forest loss. In a–b, the proportion of the landscape in forest was binned into intervals of 0.2. In c–d, forest loss (proportion of forest in the landscape in 2000 – the proportion of the landscape in forest in 2020) was binned into intervals of 0.2. The left panel shows results the original forest fragmentation index, calculated as described in Ma et al. (2023), and the right panel a modified forest fragmentation index, which included the full range of patch numbers, edge densities, and mean patch sizes rather than truncating outlier values. Fragmentation was classified as increasing when there were higher forest fragmentation index values in 2020 than in 2000. Results are for landscapes with a 1-km radius (n = 63,019); only landscapes that lost forest between 2000 and 2020 are included


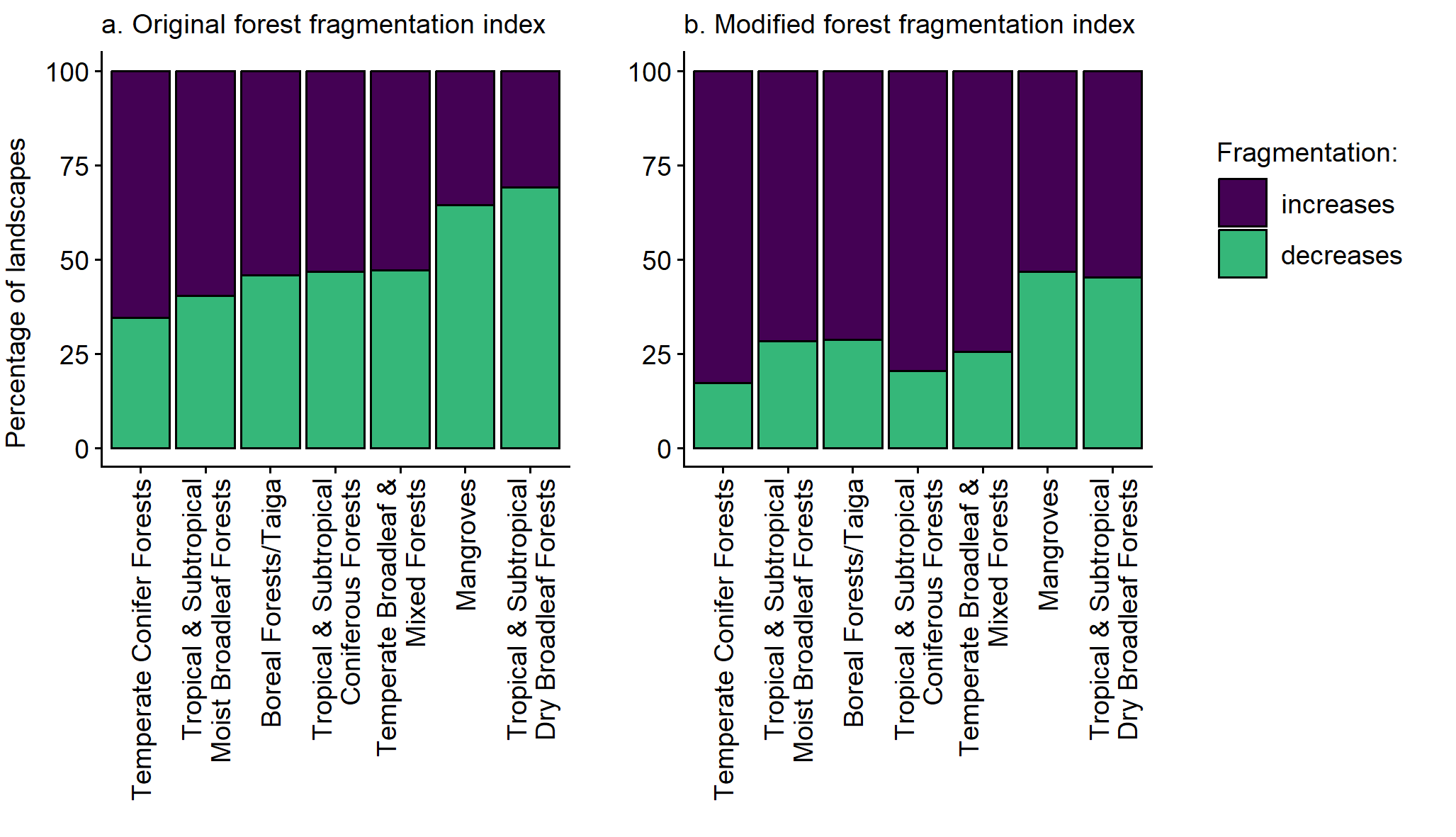


**Fig. S3** Percentages of landscapes that became more fragmented, less fragmented, and showed no change in fragmentation associated with forest loss, in each of the seven forested biomes, when using the (a) the original forest fragmentation index, calculated as described in Ma et al. (2023), and (b) a modified forest fragmentation index, which included the full range of patch numbers, edge densities, and mean patch sizes rather than truncating outlier values. Fragmentation was classified as increasing when there were higher forest fragmentation index values in 2020 than in 2000. Results are for landscapes with a 1-km radius (n = 63,019); only landscapes that lost forest between 2000 and 2020 are included
