# Supplementary material for: Habitat fragmentation can either increase or decrease with habitat loss
Source: Landsc Ecol. 2026 Apr 9;41(6):97. doi: 10.1007/s10980-026-02345-8 (PMC13194208; doi:10.1007/s10980-026-02345-8)
Supplement: Supplementary file 3 — Supplementary file3 (DOCX 249 KB) [file 10980_2026_2345_MOESM3_ESM.docx]

**Online Resource 3**


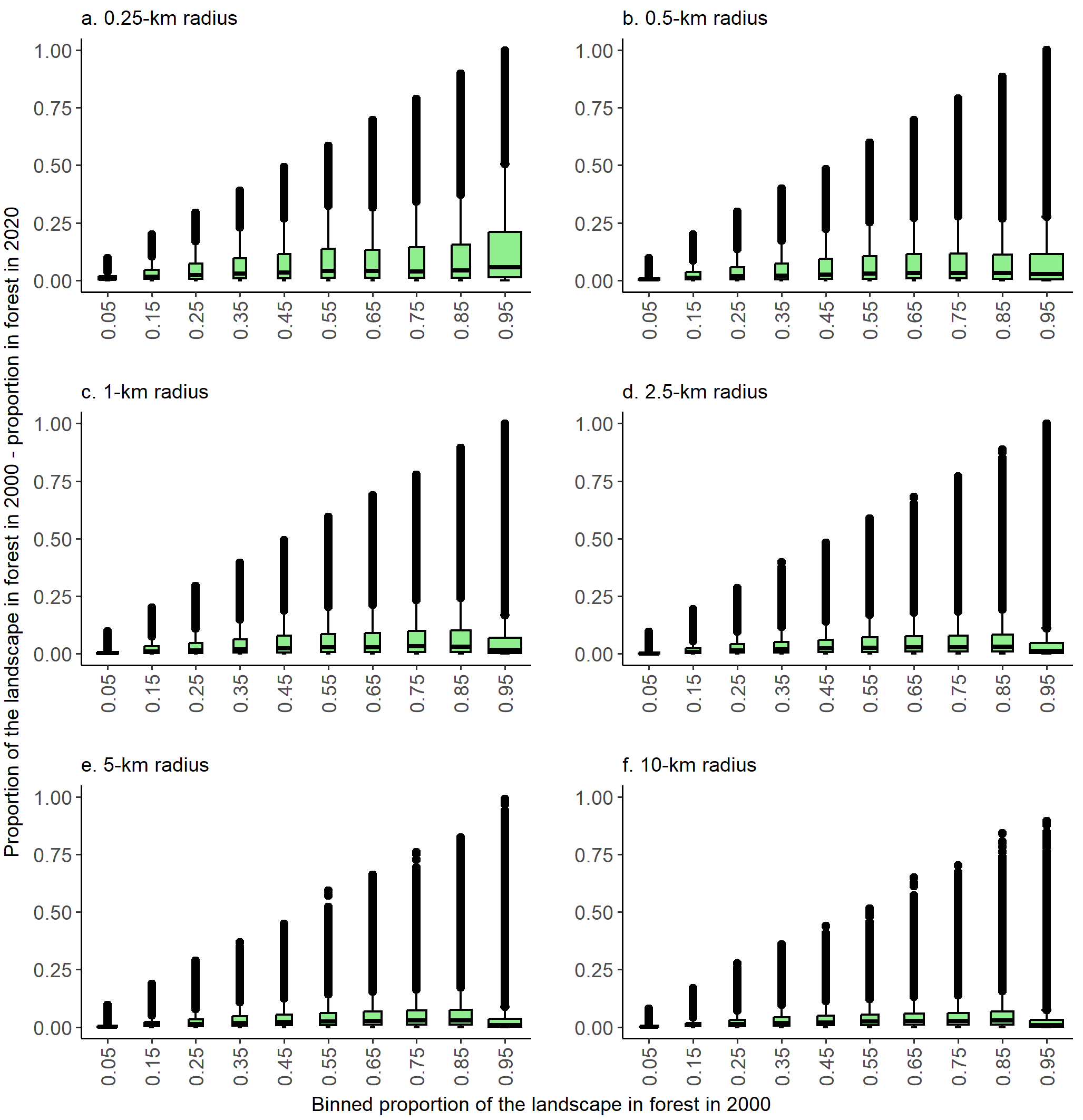


**Fig. S1** Variations in forest loss (proportion of the landscape in forest in 2000 – proportion in forest in 2020) with the proportion of the landscape in forest in 2000, for each of six landscape sizes. For visualization purposes, the proportion of the landscape in forest was binned into intervals of 0.1. Boxes = interquartile ranges, whiskers = 1.5 × interquartile ranges, horizontal lines = medians, and points = outliers. The total number of landscapes ranged from 36,482 at the smallest landscape size to 84,635 at the largest landscape size (Table S1 in Online Resource 4); only landscapes that lost forest between 2000 and 2020 are included


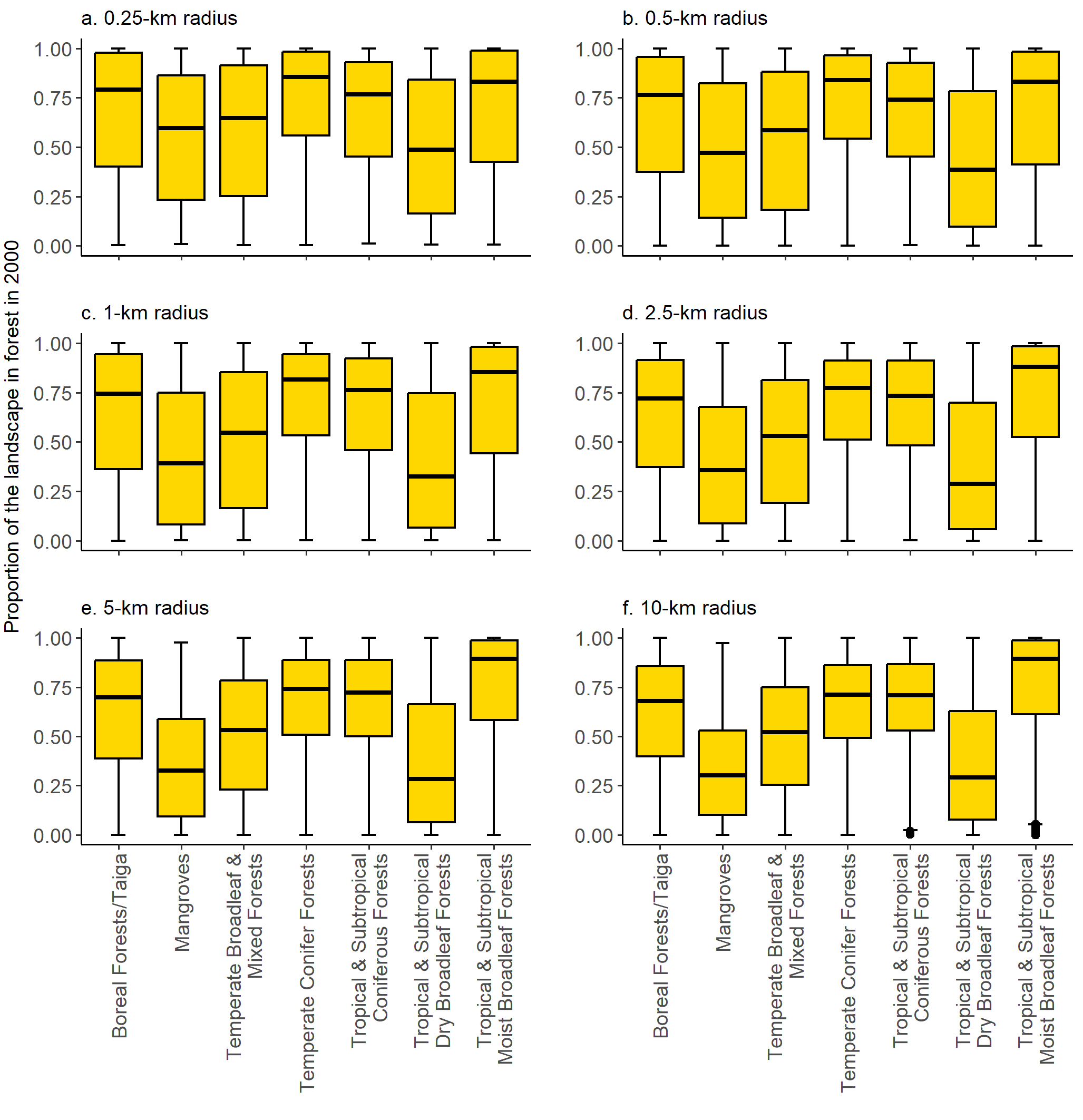


**Fig. S2** Variation in the proportion of the landscape in forest in 2000 across biomes, for each of six landscape sizes. Boxes = interquartile ranges, whiskers = 1.5 × interquartile ranges, horizontal lines = medians, and points = outliers. The total number of landscapes ranged from 36,482 at the smallest landscape size to 84,635 at the largest landscape size (Table S1 in Online Resource 4); only landscapes that lost forest between 2000 and 2020 are included


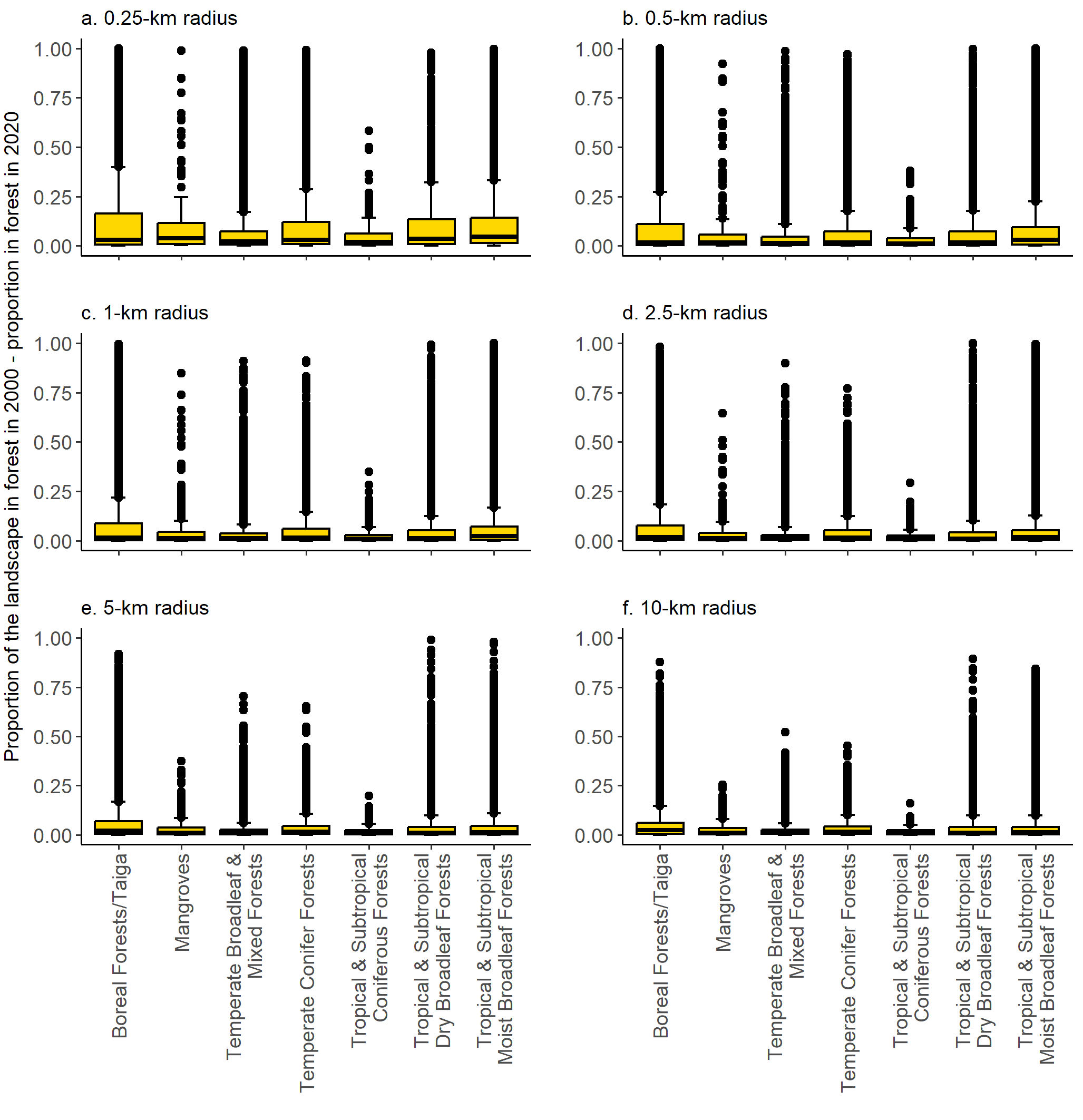


**Fig. S3** Variation in forest loss (proportion of the landscape in forest in 2000 – proportion in forest in 2020) across biomes, for each of six landscape sizes. Boxes = interquartile ranges, whiskers = 1.5 × interquartile ranges, horizontal lines = medians, and points = outliers. The total number of landscapes ranged from 36,482 at the smallest landscape size to 84,635 at the largest landscape size (Table S1 in Online Resource 4); only landscapes that lost forest between 2000 and 2020 are included
