# Supplementary material for: Habitat fragmentation can either increase or decrease with habitat loss
Source: Landsc Ecol. 2026 Apr 9;41(6):97. doi: 10.1007/s10980-026-02345-8 (PMC13194208; doi:10.1007/s10980-026-02345-8)
Supplement: Supplementary file 4 — Supplementary file4 (DOCX 336 KB) [file 10980_2026_2345_MOESM4_ESM.docx]

**Online Resource 4**


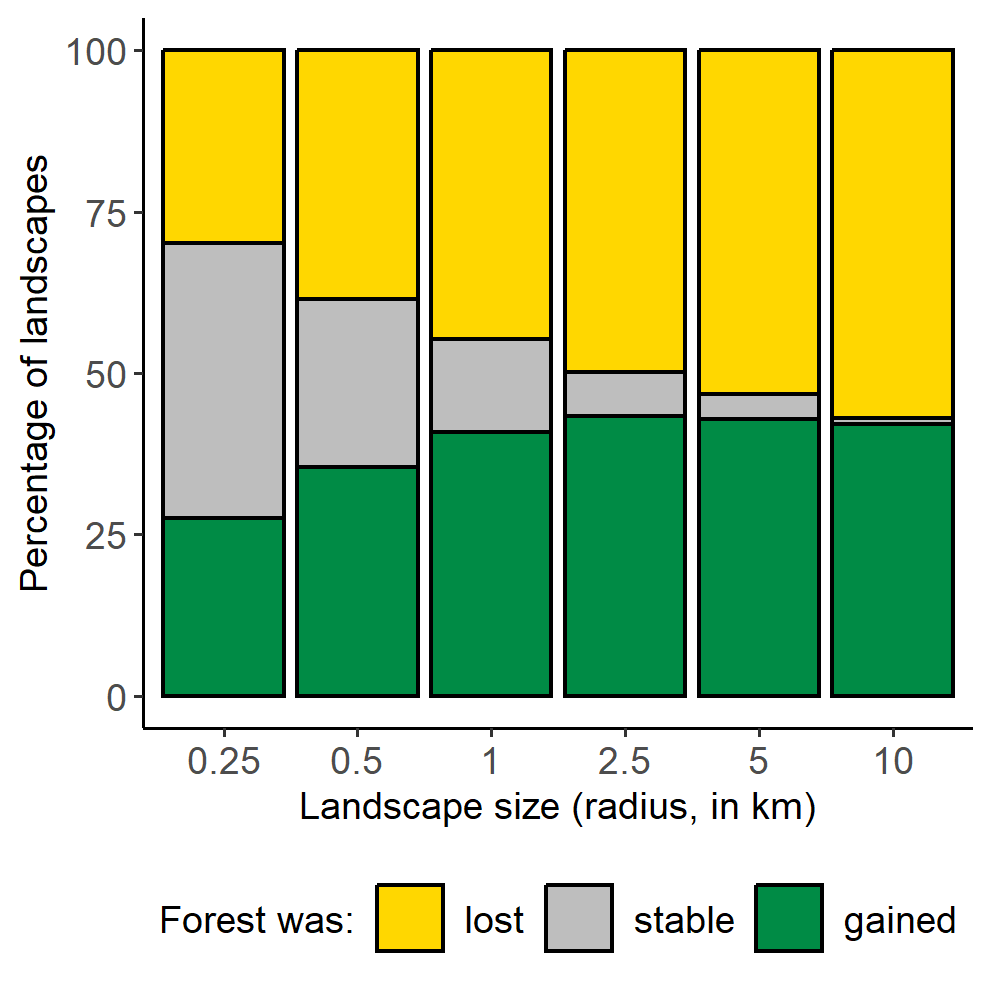


**Fig. S1** Percentage of landscapes where forest was lost, stable, or gained between 2000 and 2020, for each of the six landscape sizes. We calculated the percentage of landscapes as the number of landscapes where forest was lost (or stable or gained) / total number of landscapes × 100

**Table S1** Summary of forest amounts in 2000, forest loss between 2000 and 2020, and sample sizes for each of the six landscape sizes (reported as the radius of the circular landscape, in km)

| Landscape size | Mean (range) for the proportion of the landscape in forest in 2000 | Mean (range) for the proportion of forest lost from 2000 to 2020 | Number of landscapes |
| --- | --- | --- | --- |
| 0.25 | 0.65 (<0.01–1.00) | 0.11 (<0.01–1.00) | 36,482 |
| 0.5 | 0.63 (<0.01–1.00) | 0.08 (<0.01–1.00) | 51,392 |
| 1 | 0.62 (<0.01–1.00) | 0.07 (<0.01–1.00) | 63,019 |
| 2.5 | 0.62 (<0.01–1.00) | 0.05 (<0.01–1.00) | 72,697 |
| 5 | 0.63 (<0.01–1.00) | 0.05 (<0.01–0.99) | 78,625 |
| 10 | 0.63 (<0.01–1.00) | 0.04 (<0.01–0.89) | 84,635 |


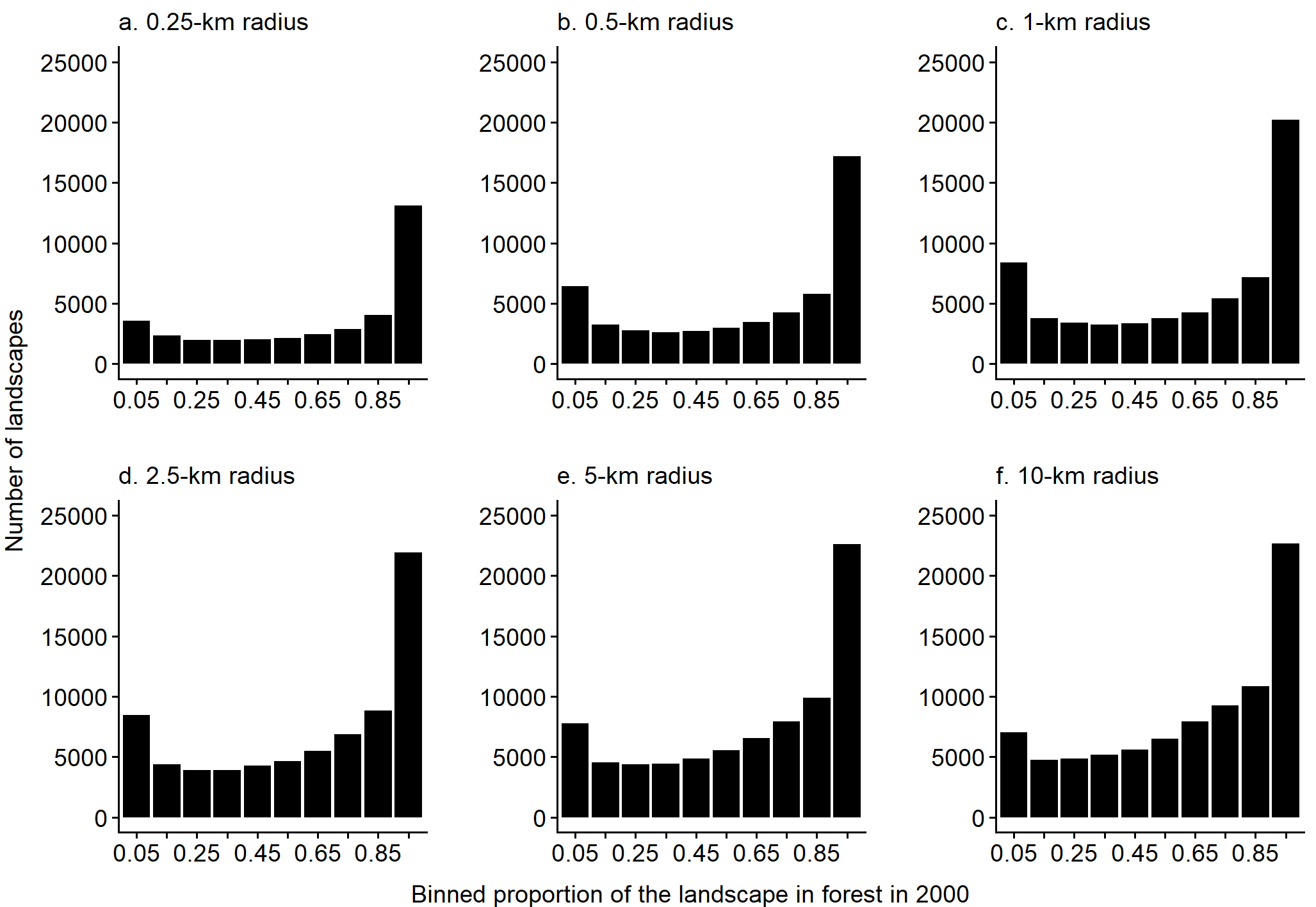


**Fig. S2** The numbers of landscapes that had different amounts of forest in 2000, for each of the six landscape sizes. The proportion of the landscape in forest in 2000 was binned into intervals of 0.1


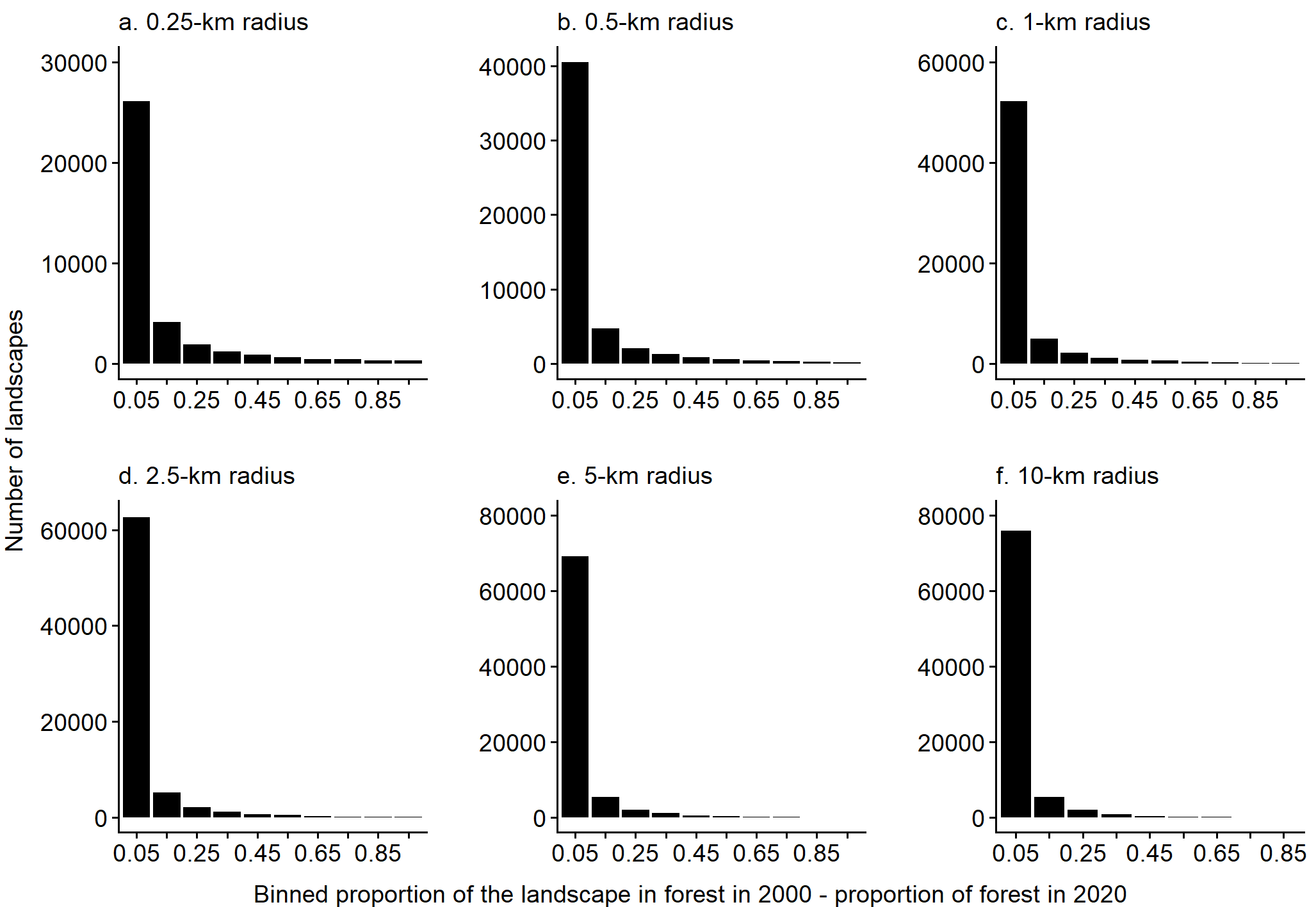


**Fig. S3** The numbers of landscapes that lost different amounts of forest, for each of the six landscape sizes. Forest loss was measured as the proportion of the landscape in forest in 2000 minus the proportion of the landscape in forest in 2020, and binned into intervals of 0.1


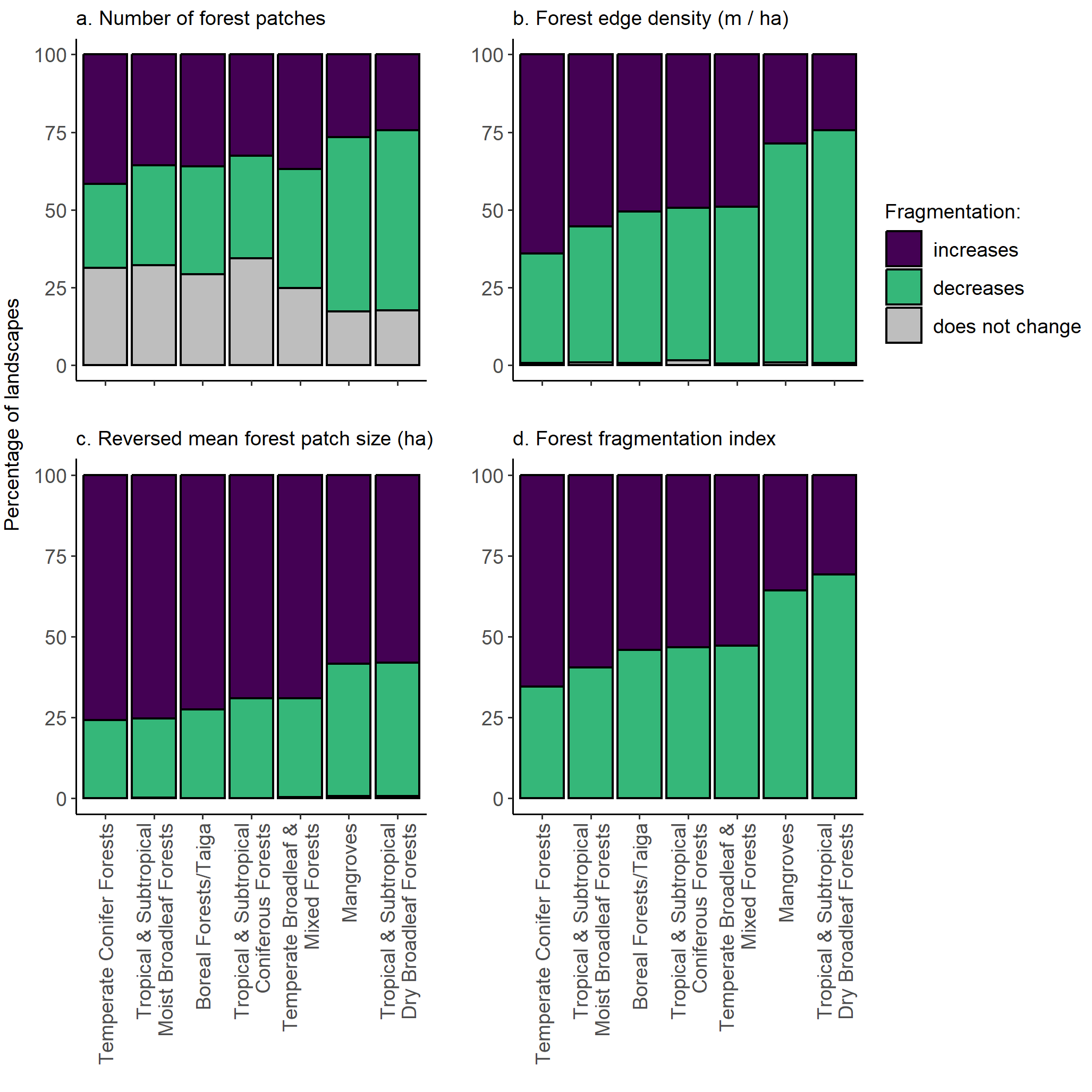


**Fig. S4** Percentages of landscapes that became more fragmented, less fragmented, and showed no change in fragmentation associated with forest loss, in each of the seven forested biomes. Fragmentation was classified as increasing when there were (a) more forest patches, (b) higher forest edge density (meters of forest–non-forest edge per ha, including all patches in the landscape), (c) smaller mean forest patch sizes, and (d) higher forest fragmentation index values in 2020 than in 2000. Results are for landscapes with a 1-km radius (n = 63,019); only landscapes that lost forest between 2000 and 2020 are included


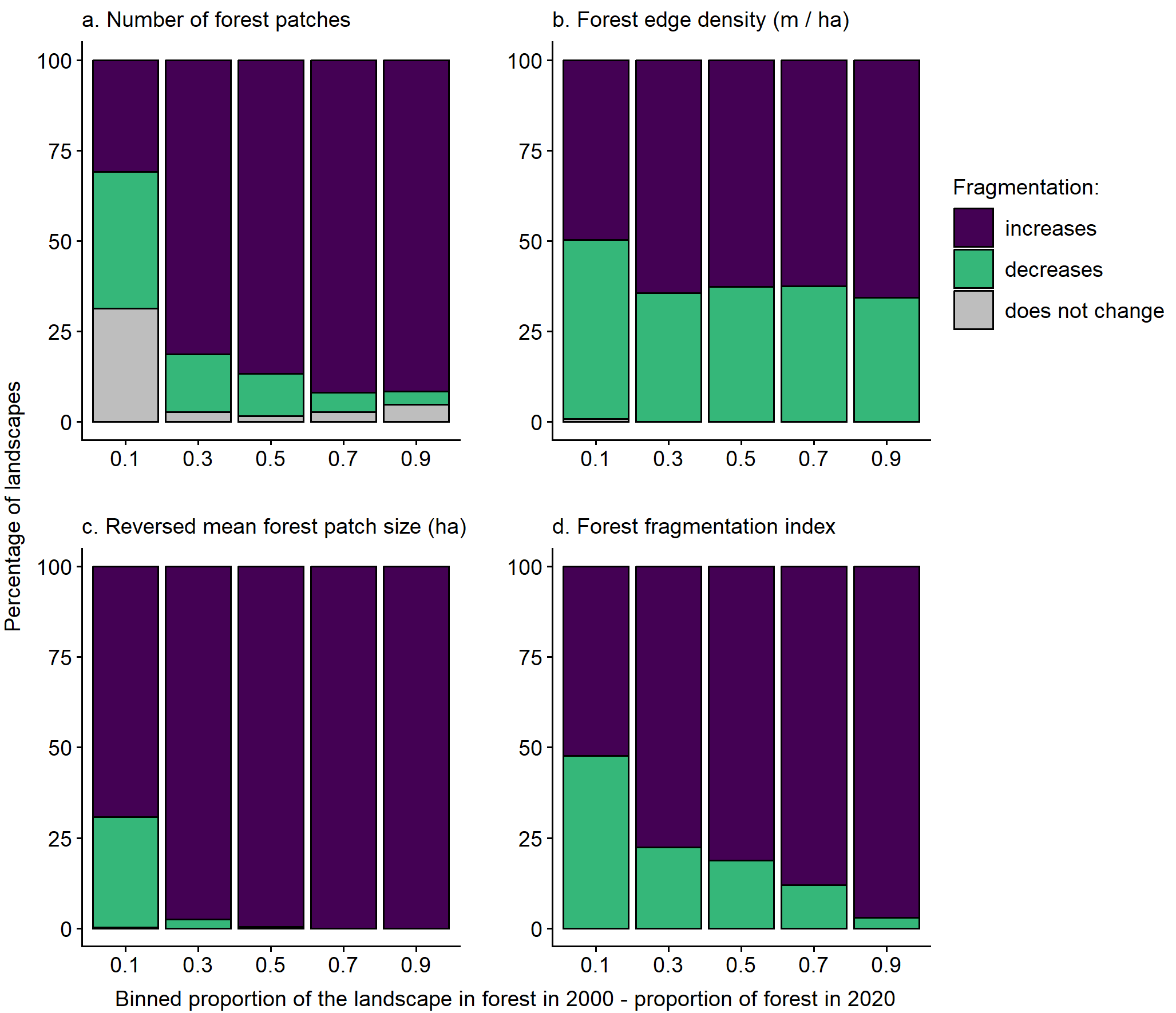


**Fig. S5** Relationships between the magnitude of forest loss from 2000 and 2020 and the percentages of landscapes that became more fragmented, less fragmented, and showed no change in fragmentation associated with forest loss. Forest loss was binned into intervals of 0.2, where forest loss was the proportion of the landscape in forest in 2000 minus the proportion of the landscape in forest in 2020. Fragmentation was classified as increasing when there were (a) more forest patches, (b) higher forest edge density (meters of forest–non-forest edge per ha, including all patches in the landscape), (c) smaller mean forest patch sizes, and (d) higher forest fragmentation index values in 2020 than in 2000. Results are for landscapes with a 1-km radius (n = 63,019); only landscapes that lost forest between 2000 and 2020 are included


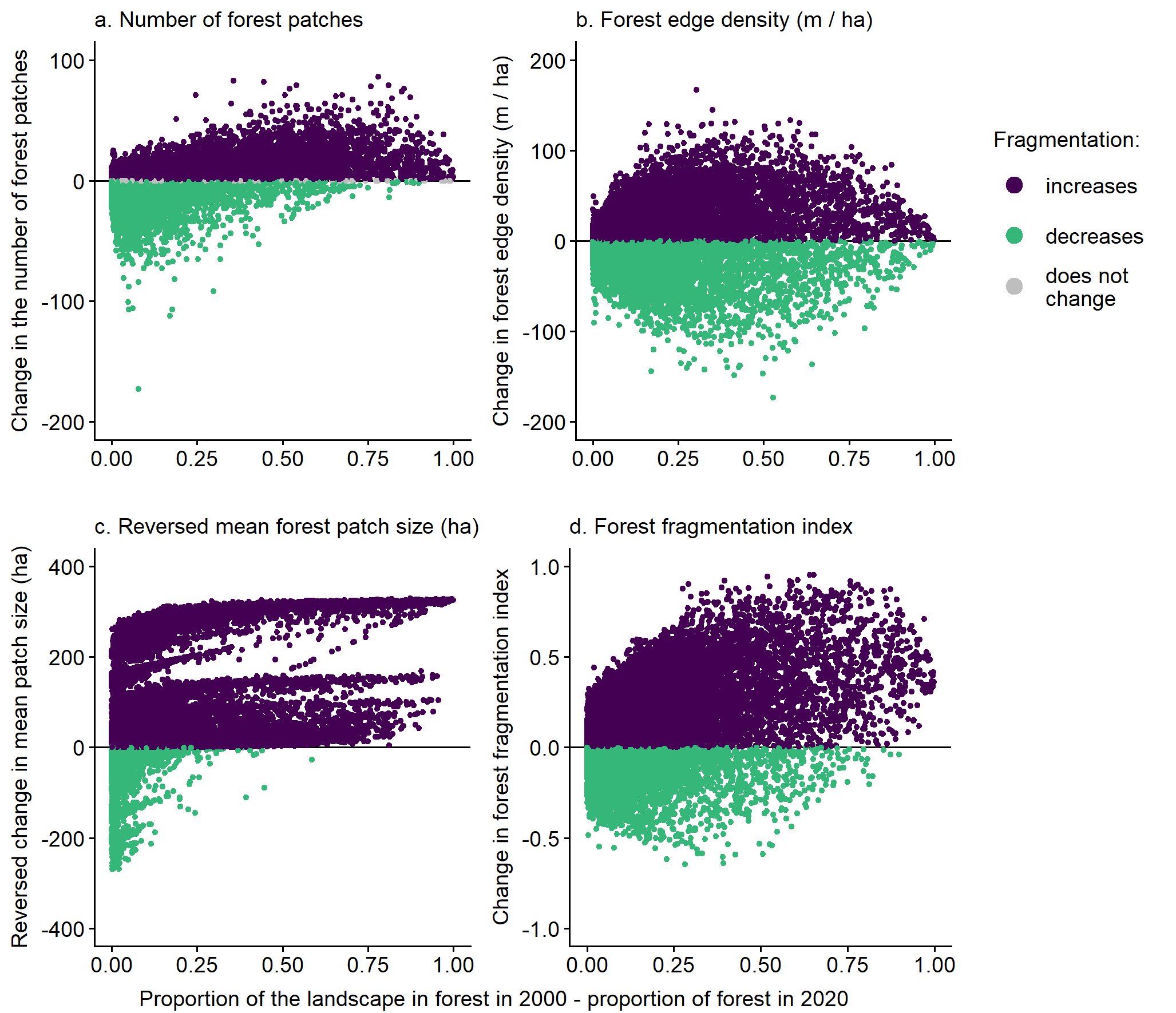


**Fig. S6** Relationships between the magnitude of forest loss and the change in forest fragmentation from 2000 to 2020, for each of four measures of forest fragmentation. Forest loss was measured as the proportion of the landscape in forest in 2000 minus the proportion of the landscape in forest in 2020. An increase in fragmentation is indicated by (a) more forest patches, (b) higher forest edge density (meters of forest–non-forest edge per ha, including all patches in the landscape), (c) smaller mean forest patch sizes, and (d) higher forest fragmentation index values in 2020 than in 2000. We plot the reversed change in mean patch size, so that increased fragmentation is associated with positive values across all metrics. Results are for landscapes with a 1-km radius (n = 63,019); only landscapes that lost forest between 2000 and 2020 are included
