# Supplementary material for: Habitat fragmentation can either increase or decrease with habitat loss
Source: Landsc Ecol. 2026 Apr 9;41(6):97. doi: 10.1007/s10980-026-02345-8 (PMC13194208; doi:10.1007/s10980-026-02345-8)
Supplement: Supplementary file 6 — Supplementary file6 (DOCX 385 KB) [file 10980_2026_2345_MOESM6_ESM.docx]

**Online Resource 6**


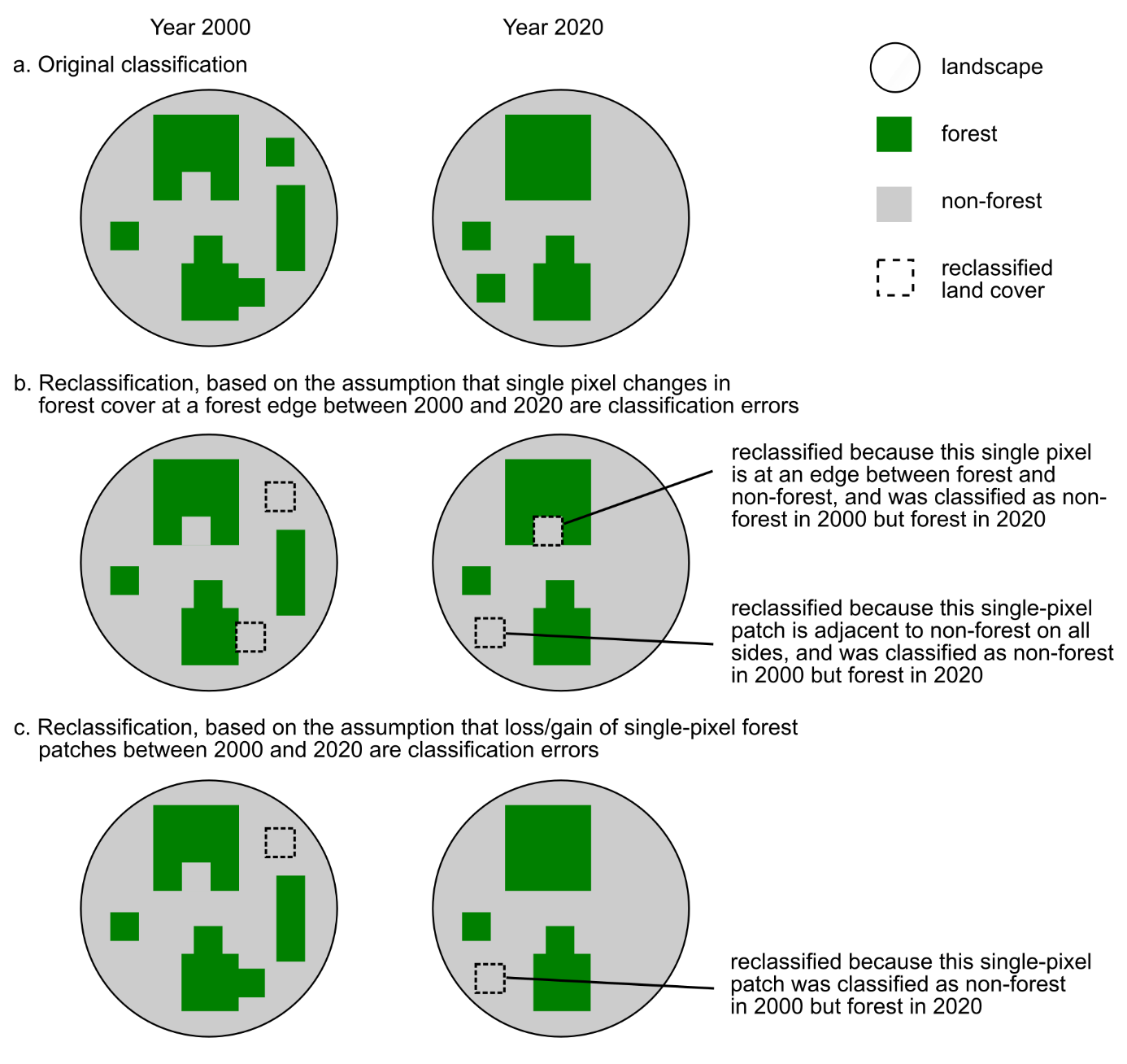


**Fig. S1** Illustrative examples to show how we reclassified land cover to assess the potential impacts of classification errors on estimates of forest loss and fragmentation. First, we assessed classification uncertainty at boundaries between forest and other land cover classes. This could, for example, cause spurious detection of changes in forest amount and fragmentation between 2000 and 2020, if that uncertainty leads to pixels at forest edges being erroneously classified as forest in one year and correctly classified as non-forest in the other year. If a change in forest cover (from forest to non-forest, or vice versa) between 2000 and 2020 occurred at a location at the edge of a forest patch and that change was a single pixel, it was reclassified as non-forest in both years before measuring changes in forest amount and fragmentation (compare panels a vs. b). Second, classification of single, isolated pixels as forest may be more likely to reflect classification errors than larger, contiguous groups of pixels. This could, for example, cause spurious detection of changes in forest cover and fragmentation if the isolated pixel is erroneously classified as forest in one year and correctly classified non-forest in the other year. We assessed this by reclassifying pixels as non-forest if a forest patch comprised of a single pixel was lost (or gained) between 2000 and 2020 (compare panels a vs. c). These reclassifications were repeated, replacing the focus on single pixels with groups of up to 2, 3, or 4 adjacent pixels


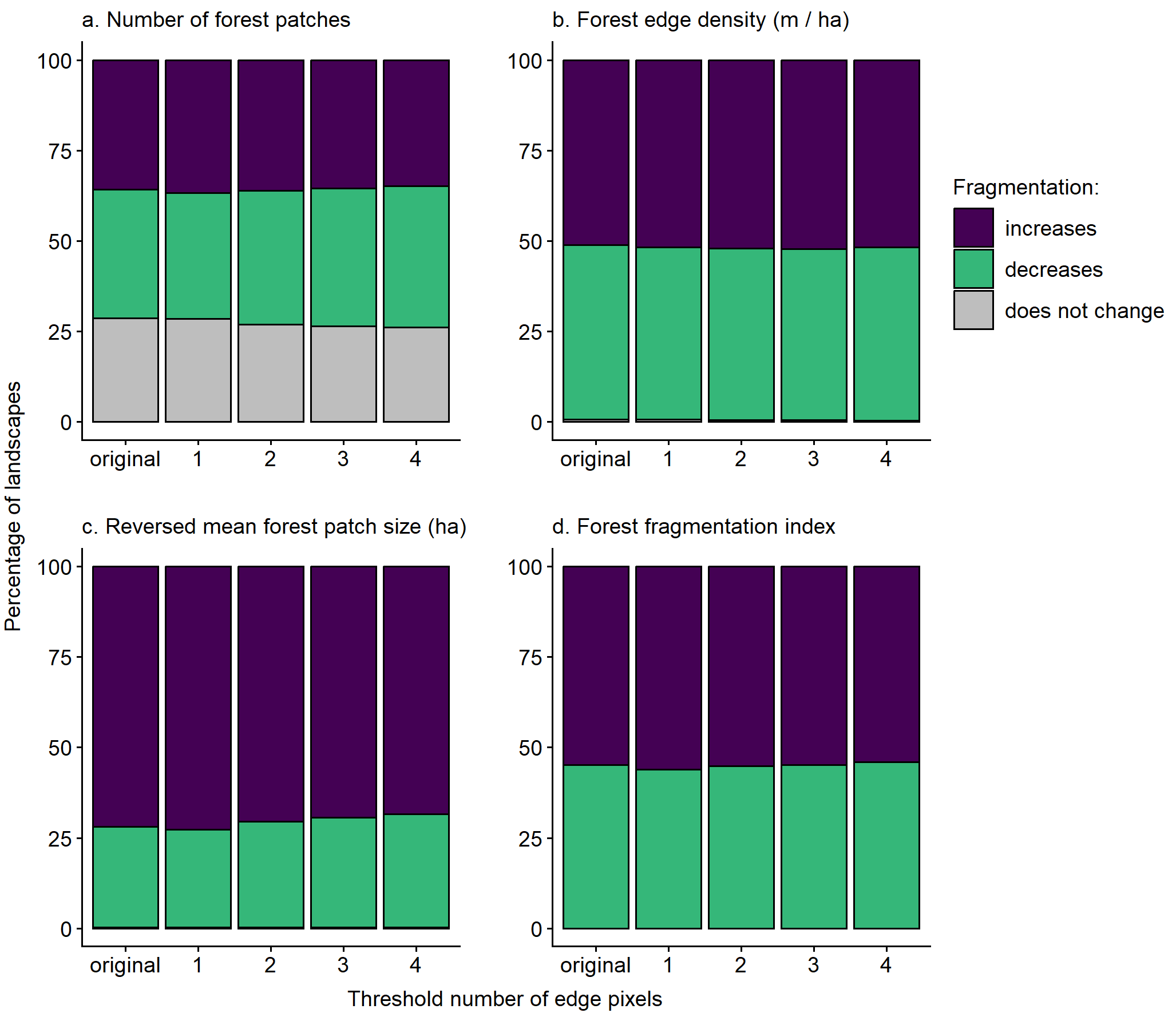


**Fig. S2** Percentages of landscapes with forests that became more fragmented, less fragmented, and showed no change in fragmentation after forest loss, when applying different thresholds for treating changes in forest cover at forest patch edges as classification error rather than true change. The first bar in each panel reflects the results when we used the original land cover classification of Potapov et al. (2022). A threshold of one indicates that, if a change in forest cover (from forest to non-forest, or vice versa) between 2000 and 2020 occurred at a location at the edge of a forest patch and that change was a single pixel, it was reclassified as non-forest in both years. Thresholds of 2 to 4 indicate that groupings of adjacent edge pixels of ≤2 to ≤4 pixels were reclassified. Fragmentation was classified as increasing when there were (a) more forest patches, (b) higher forest edge density (meters of forest–non-forest edge per ha, including all patches in the landscape), (c) smaller mean forest patch sizes, and (d) higher forest fragmentation index values in 2020 than in 2000. Results are for landscapes with a 1-km radius; only landscapes that lost forest between 2000 and 2020 are included


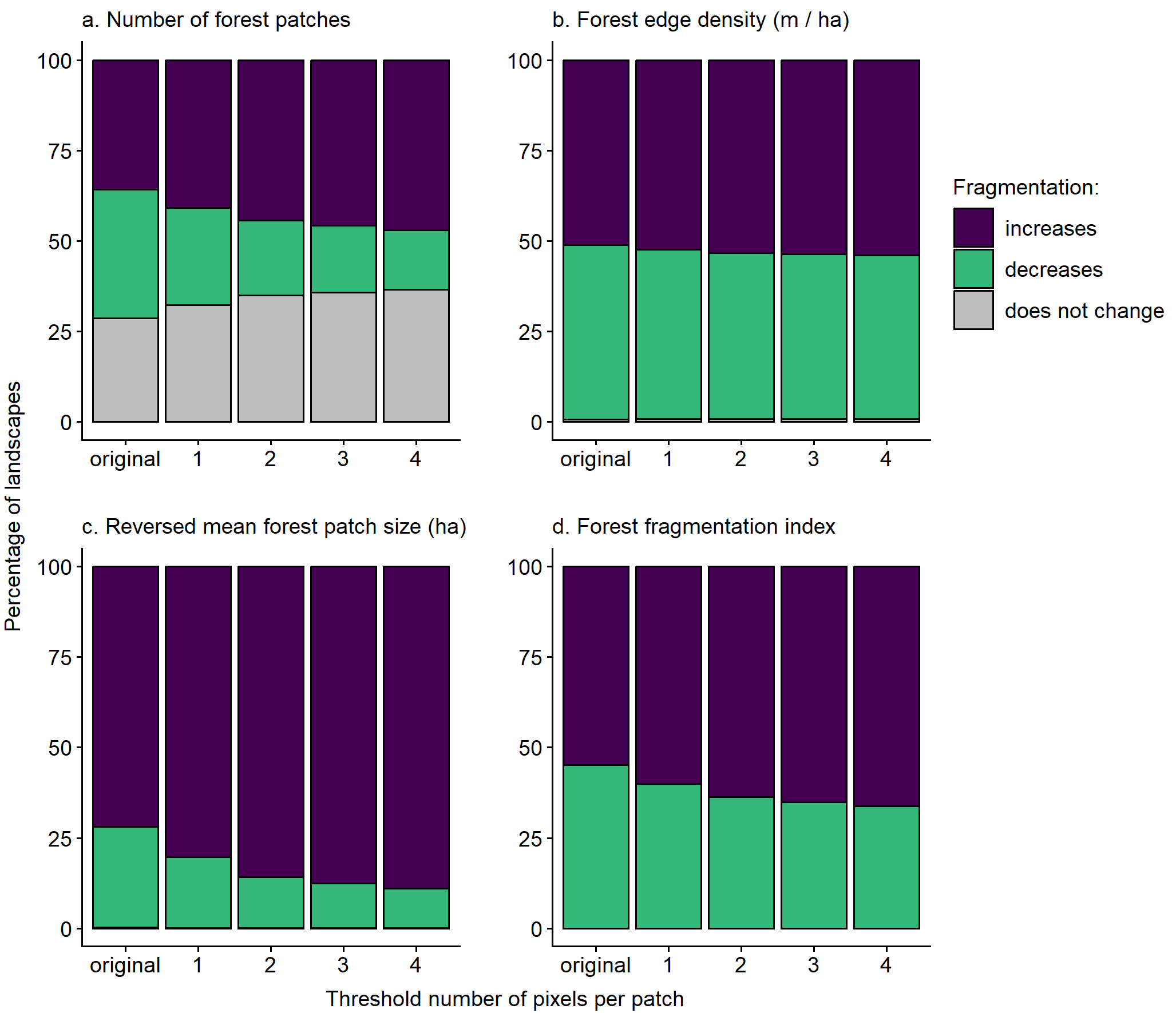


**Fig. S3** Percentages of landscapes with forests that became more fragmented, less fragmented, and showed no change in fragmentation after forest loss, when applying different thresholds for treating the loss/gain of forest patches as classification error rather than true change. The first bar in each panel reflects the results when we used the original land cover classifications of Potapov et al. (2022). A threshold of one indicates that, if a forest patch comprised of a single pixel was lost (or gained) between 2000 and 2020, it was considered a classification error and was reclassified as non-forest in both years. Thresholds of 2 to 4 indicate that groupings of adjacent edge pixels of ≤2 to ≤4 pixels were reclassified. Fragmentation was classified as increasing when there were (a) more forest patches, (b) higher forest edge density (meters of forest–non-forest edge per ha, including all patches in the landscape), (c) smaller mean forest patch sizes, and (d) higher forest fragmentation index values in 2020 than in 2000. Results are for landscapes with a 1-km radius
