# Supplementary material for: Habitat fragmentation can either increase or decrease with habitat loss
Source: Landsc Ecol. 2026 Apr 9;41(6):97. doi: 10.1007/s10980-026-02345-8 (PMC13194208; doi:10.1007/s10980-026-02345-8)
Supplement: Supplementary file 7 — Supplementary file7 (DOCX 40 KB) [file 10980_2026_2345_MOESM7_ESM.docx]

**Online Resource 7**


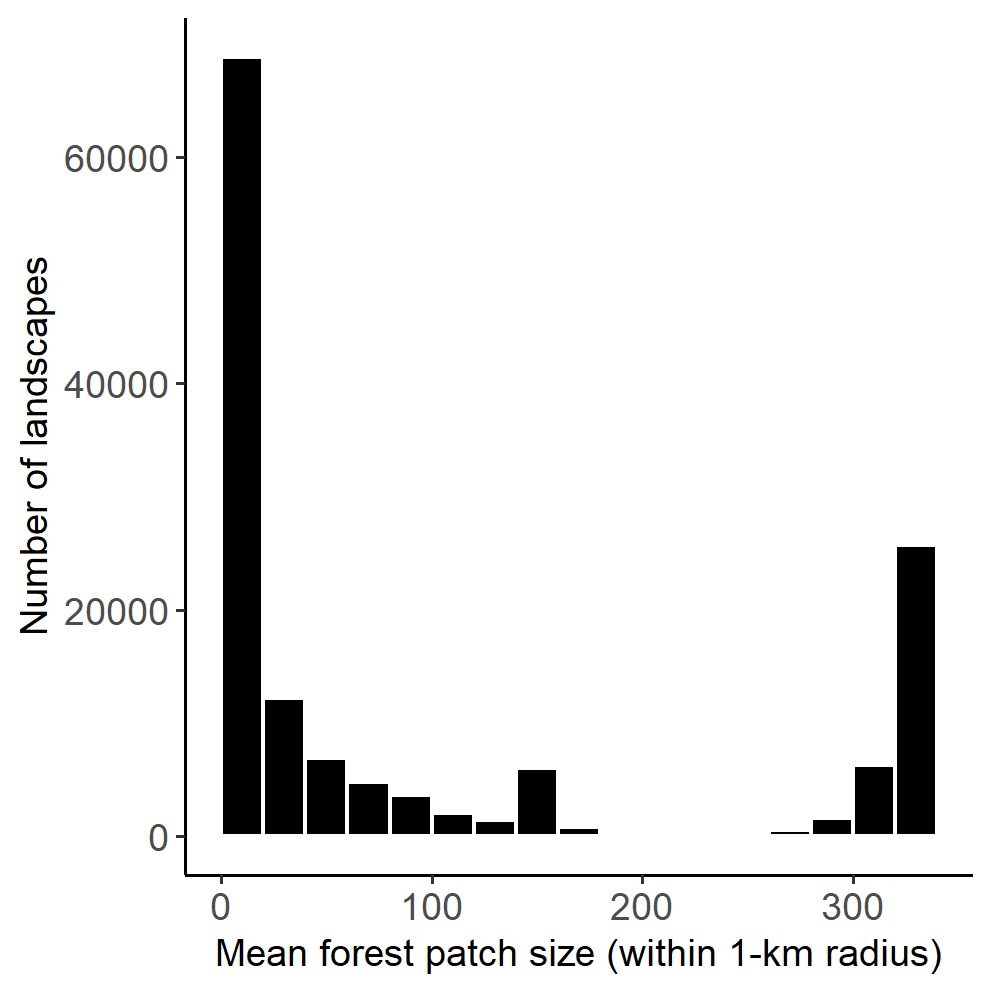


**Fig. S1** Frequency distribution of mean forest patch sizes (ha) in the year 2020, for the 141, 038 sampled landscapes with forest cover within a 1-km buffer. This includes landscapes that lost, gained, or had no change in forest cover from 2000 to 2020. 48.8% of landscapes had mean patch sizes <20 ha (i.e., fell within the first bar of the histogram)
